# The Effects of Hybridization and Parasite Infection on the Survival and Behaviour of Endangered Landlocked Salmon Subject to Predation—Implications for Genetic Rescue

**DOI:** 10.1111/eva.70056

**Published:** 2024-12-13

**Authors:** Aslak Eronen, Matti Janhunen, Pekka Hyvärinen, Raine Kortet, Anssi Karvonen

**Affiliations:** ^1^ Department of Environmental and Biological Sciences University of Eastern Finland Joensuu Finland; ^2^ Natural Resources Institute Finland (LUKE) Migratory Fish and Regulated Rivers Joensuu Finland; ^3^ Natural Resources Institute Finland (LUKE) Migratory Fish and Regulated Rivers Paltamo Finland; ^4^ Department of Biological and Environmental Science University of Jyväskylä Jyväskylä Finland

**Keywords:** conservation biology, genetic rescue, host parasite interactions, hybridization, outbreeding depression, *salmo salar* m. *sebago*

## Abstract

A prerequisite of genetic rescue in endangered and genetically depauperate populations is to pre‐evaluate between possible pros and cons of hybridization for the life history and survival of the target population. We hybridized the critically endangered Saimaa landlocked salmon ( *Salmo salar* m. *sebago*) with one of its geographically closest relatives, anadromous Baltic salmon from River Kymijoki. In two similar experiments, conducted in semi‐natural streams during overwintering (at age 1.5) and in early summer (age 2+), we studied how hybridization and eye parasite infection (*Diplostomum pseudospathaceum*) affected survival from predation by Northern pike ( *Esox lucius* ). Additionally, we recorded movements of the juvenile salmon using passive integrated telemetry to gain insights into the effect of hybridization and infection on antipredatory behaviour (movement activity and habitat use). Among the uninfected groups, we found significantly lower mortality of hybrid salmon (mortality ± S.E. 14.5% ± 5.4%) compared to purebred landlocked salmon (37.2% ± 9.4%), supporting a positive effect of hybridization under predation risk. This benefit, however, was cancelled out by the parasite infection, which impaired vision and increased the susceptibility to predation. The negative effects of infection were particularly pronounced in the anadromous salmon due to lower infection resistance, compared to the landlocked salmon. Hybridization per se did not affect the activity levels of salmon, but overwintering activity correlated positively with eye cataract coverage, and summer activity was highest in anadromous salmon. These results demonstrate that controlled supplementation of a small animal population with genetically more diverse hybrids could entail both positive and negative implications, at least in the first crossbred generation.

## 1 Introduction

Freshwater fish diversity worldwide is increasingly threatened by anthropogenic changes in aquatic environments (Limburg and Waldman 2009; Arthington et al. 2016). In particular, migratory fish species have suffered from overfishing and habitat destruction, such as damming of rivers for hydroelectric power (Limburg and Waldman 2009; Arthington et al. 2016; Costa et al. 2021). These actions often result in fragmentation of the population structure and reduction in population size, thus increasing the probability of reduced genetic diversity and inbreeding. Genetic rescue, the increase of population fitness through immigration of conspecifics harbouring higher genetic polymorphism (i.e., gene flow), is increasingly recognized in conservation biology as a countermeasure against the negative effects of small population size (Tallmon, Luikart, and Waples 2004; Whiteley et al. 2015; Ralls et al. 2018; Bell et al. 2019). One mechanism that can lead to genetic rescue is through masking of harmful recessive alleles that may have accumulated in small populations because of random genetic drift and inbreeding. Further, introducing new genetic material into a genetically depauperate population may also increase the overall fitness and adaptive potential, as heterozygosity within the population increases. Previous theoretical considerations of genetic rescue are also supported by empirical examples, where small and inbred populations have successfully been saved from the brink of extinction by introducing individuals from larger, genetically more diverse populations (e.g., Westemeier et al. 1998; Madsen et al. 1999; Johnson et al. 2010).

While the theory underlying genetic rescue is straightforward, the effect of interplay between genetics and ecology on fitness in natural populations may manifest via complex interactions. For instance, the well‐known example of genetic rescue of the Florida panther also involved inadvertent transferral of a new form of feline immunodeficiency virus from Texas to Florida (Johnson et al. 2010; Malmberg et al. 2019). Since pathogens and parasites have profound effects on fitness, survival, and evolution of their hosts, accidental spread of infections during genetic rescue actions can lead to unintended outcomes (Currens et al. 1997; Barber, Hoare, and Krause 2000; Goldberg et al. 2005; Malmberg et al. 2019). Further, when different populations hybridize as a consequence of genetic rescue, also their heritable resistance to different pathogens and parasites, whether introduced or already present in the population, can be affected (Currens et al. 1997; Goldberg et al. 2005; Heber et al. 2013; Klemme et al. 2021; Lewis et al. 2023). For example, genetic rescue and the increase in genetic diversity can improve host immune function relative to the original inbred population (Heber et al. 2013; Klemme et al. 2021; Lewis et al. 2023). On the other hand, outbreeding depression following the genetic mixing can result in increased susceptibility to certain infections (Currens et al. 1997; Goldberg et al. 2005; Klemme et al. 2021). Despite of such profound potential effects on the outcome of genetic rescue actions, the role of pathogens and parasites has often been neglected in genetic rescue studies. At the same time, other ecological interactions, such as foraging and predator avoidance, have received more attention (Houde, Fraser, and Hutchings 2010; Ågren et al. 2019; Eronen et al. 2023).

Some effects of hybridization may not be seen before later generations, when recombination among hybrid backcrosses leads to new genetic combinations, some of which may be beneficial and others harmful (Gharrett et al. 1999; Frankham 2016; Bell et al. 2019). Additionally, positive and negative effects are not mutually exclusive; while the negative effects of inbreeding may be reversed in some traits, outbreeding depression might occur in others (Eronen et al. 2021; Klemme et al. 2021, 2024). In light of these complex interactions, possible fitness benefits of genetic rescue should always be carefully weighted against the possible disadvantages of hybridization (Burridge 2019; Bell et al. 2019). This is especially important if there are no closely related populations available as donors since the risk of outbreeding depression increases with genetic and ecological distance between the donor and recipient populations (Pekkala et al. 2012; Frankham 2015; Kronenberger et al. 2017).

Among freshwater fishes, one population that could benefit from genetic rescue actions is the critically endangered landlocked salmon ( *Salmo salar* m. *sebago* Girard, 1853) native to the Vuoksi watershed (Lake Saimaa) in Eastern Finland (Urho et al. 2019; Eronen et al. 2021, 2023). A hatchery program aiming to conserve this unique Atlantic salmon population was started in the 1960s when nearly all the original spawning and nursery habitats were harnessed for hydroelectric power production (Pursiainen, Makkonen, and Piironen 1998; Hutchings et al. 2019). Thereafter, the population has been maintained almost exclusively by annual stocking of hatchery‐raised 2‐ or 3‐year‐old smolts, some of which return to their stocking site as adults. Collecting eggs and milt from these returning spawners is an essential part in maintaining the population to incorporate natural selection in at least part of the life‐history, reducing the potential for domestication in the population (Janhunen, Turkka, and Kekäläinen 2023). Following the collapse of the natural population, the landlocked salmon underwent severe genetic bottlenecks, and the effective population size has remained at very low level ever since (Koljonen et al. 2002; Hutchings et al. 2019). Heterozygosity in the landlocked salmon, based primarily on microsatellite data, is currently considerably lower (0.28–0.47) than in geographically close Lake Ladoga landlocked populations (0.49–0.60) or in anadromous populations (0.60–0.75) of the Baltic Sea (Koljonen et al. 2002; Tonteri et al. 2005; Ozerov et al. 2010). Therefore, controlled introductions of hybrid individuals to this genetically depauperate population could possibly be used as the last option to revive the population and prevent extinction. Because only a tiny fraction of the stocked landlocked salmon survive the feeding migration to be recaptured as spawners, even small improvements in post‐stocking survival could be of high significance in maintaining the remaining genetic variation within the population. The landlocked salmon of Lake Saimaa has, however, been reproductively isolated from its closest conspecifics for 5700–10 200 years (i.e., 950–1700 generations) (Berg 1985; Tikkanen and Mäkiaho 2012; Lumme et al. 2015). Therefore, hybridisation of these populations can raise concerns of possible outbreeding depression (Frankham et al. 2011; Bell et al. 2019).

Eye flukes of the genus *Diplostomum* are common parasites in freshwater and brackish water fishes (Voutilainen et al. 2009; Kuukka‐Anttila et al. 2010; Seppälä, Karvonen, and Valtonen 2011) and prevalent also in fish hatcheries producing landlocked salmon smolts for stocking (Kuukka, Peuhkuri, and Kolari 2006; Seppänen et al. 2008). Valtonen and Gibson (1997), for example, found *Diplostomum* species in 21 of the 25 studied fish species in northern Finland, while Voutilainen et al. (2009) found them in 22 out of 28 studied Finnish lakes and ponds. These parasites reproduce asexually in gastropods, use fish as intermediate hosts (certain species infecting the eye lenses), and mature in the intestine of fish‐eating birds (Chappell, Hardie, and Secombes 1994; Chappell 1995). Infection in the eye lenses causes cataracts and the resulting impairment of vision of fish can make them more susceptible to predation both by avian definitive hosts (Crowden and Broom 1980; Seppälä, Karvonen, and Valtonen 2004, 2005) and non‐host predatory fish (Karvonen et al. 2023). Interestingly, the landlocked salmon is more resistant to *D. pseudospathaceum* (Niewiadomska 1984) than geographically close Baltic anadromous populations (Klemme et al. 2021; Klemme et al. 2024; but see also Seppänen et al. 2008). Consequently, hybrids of these populations have lower resistance against this parasite (Klemme et al. 2021), undermining the possible benefits of hybridization (i.e., outbreeding depression). Furthermore, Klemme et al. (2024) found that *D. pseudospathaceum* infection increased susceptibility of the hybrids (albeit not statistically significantly) to piscine predation compared to landlocked salmon at age 1+. The consequences of hybridization and infection with *D. pseudospathaceum* for salmon survival through other critical life‐history stages, for example, the overwintering period or later, have not been examined.

In the present study, we hybridized the critically endangered landlocked salmon of the Vuoksi watershed with a geographically close Baltic anadromous salmon. Thereafter, we conducted two experiments in semi‐natural conditions to examine growth, predation‐induced mortality, and behavioural traits among the crossing groups. These experiments were conducted at two life stages: during overwintering (at age 1.5–2 years) and in early summer (at age 2+). Northern pike ( *Esox Lucius* Linnaeus 1758), a common piscine predator of juvenile salmonids (e.g., Kekäläinen, Niva, and Huuskonen 2008), was used as a predator. In both experiments (winter and summer), half of the salmon were infected with *D. pseudospathaceum* to explore how parasitism in a central sensory organ influenced the crossing groups. Passive integrated telemetry (PIT) antennae were applied to record the behaviour of individuals (i.e., activity and habitat use), both in the presence and absence of predators. These data were collected to gain insights into mechanisms underlying possible survival differences among groups (previous studies have indicated behavioural differences between the crossing groups; Eronen et al. 2023; Klemme et al. 2024).

Our first hypothesis was that hybridization could improve survival from predation relative to the landlocked salmon. Our previous study showed a higher tendency for explorative behaviour and lower stress response in landlocked salmon compared to anadromous salmon, with intermediate values in hybrids (Eronen et al. 2023). Both behavioural features were linked to elevated boldness in the landlocked salmon, which again suggests that hybrids could be more cautious in the presence of predators. Second, we expected that the possible positive effects of hybridization could be removed by infection with *D. pseudospathaceum*. This is because hybrids have reduced resistance towards the infection compared to landlocked salmon (Klemme et al. 2021) and, consequently, they could suffer more from parasite‐induced cataracts. Following the earlier work, we also expected parasitism and predation to reduce growth and activity of the fish (Karvonen et al. 2023; Klemme et al. 2024).

## 2 Materials and Methods

The study was conducted at the Kainuu Fisheries Research Station in Paltamo, Finland (64°24′N 27°31′ E) maintained by the Natural Resources Institute Finland (Luke). The station is a flow‐through facility that takes water from the nearby water body, Lake Kivesjärvi. Thus, water chemistry and temperature follow that of the source lake (mixture of water taken from the surface and hypolimnion) and are the same for all experimental tanks within the facility. The research station, however, is located in a different water system than the original living areas of the studied salmon populations.

### 2.1 Experimental Fish

In vitro fertilizations were performed among the landlocked salmon from the Vuoksi watershed (*LS*) and the Baltic anadromous salmon from the River Kymijoki (*AS*, the strain introduced originally from the River Neva, North‐Western Russia). The *LS* parents were wild‐caught spawners from the River Pielisjoki (62°42' N 29°52' E), which had been stocked there as smolts and returned to their stocking site as sexually matured adults. The *AS* parents represented a hatchery broodstock maintained at Laukaa Fisheries Research and Aquaculture station (62°28' N 25°52' E) descending from wild‐caught spawners of River Kymijoki (60°31' N 26°51' E).

The pairwise matings were conducted in October 2018 using 15 males and 15 females from both salmon populations, resulting in four crossing groups: purebred landlocked salmon (*LS × LS*), landlocked salmon females crossed with anadromous salmon males (*LS × AS*), anadromous salmon females crossed with landlocked salmon males (*AS × LS*), and purebred anadromous salmon (*AS × AS*). One part of the eggs from each female was fertilized with one male from its own population and the other part with one male from the other population. Correspondingly, equal portions of milt from each male were used to fertilize eggs of one female from its own population and of one female from the other population (*n =* 60 full‐sibling families, 15 per crossing group). To avoid inbreeding within the purebred AS families, they were produced by using parents from separate year classes 2011 and 2014. For purebred landlocked salmon, possible inbreeding could not be fully controlled as the genetic relationships of wild‐caught spawners were not investigated prior to matings. However, as landlocked males usually reach maturity at a later age than females, direct inbreeding can be mostly avoided by random mating.

After fertilization, the crossing groups were incubated separately but under identical conditions in flow‐through trays. After hatching in May 2019, the crossing groups were reared separately in eight 3.2 m^2^ fiberglass tanks (two replicate tanks per crossing group) until the fish could be individually marked. On 20th February 2020, 2000 salmon sampled evenly from each crossing group and rearing tank were tagged with 2 × 12 mm passive integrated transponders (HDX+ PIT‐tags, Biomark), enabling their individual identification. More fish were marked than needed as they were used also in other experiments. Thereafter, all the experimental fish were combined into a large (15 m^2^) tank to await the experiments.

### 2.2 Parasite Exposure

On July 2nd 2020, 960 tagged fish, 240 from all crossing groups, were sampled and exposed to *D. pseudospathaceum* (*n* = 480, 120 per cross) or sham‐exposed (*n* = 480). Again, more fish than needed were exposed to act as a reserve for the present experiment and to be used in other experiments. Infective stages (cercariae) of the parasite were obtained from 20 naturally infected *Lymnaea stagnalis* snails (first intermediate host of the parasite) collected from Lake Konnevesi (62°36' N, 26°33' E) and Lake Veijonjärvi (61°57' N, 25°41' E) 1 week before the exposures and maintained in individual containers with lake water at +4°C. It is important to note that *D. pseudospathaceum* does not show a detectable genetic population structure in the snail host across a large geographical scale (Louhi et al. 2010), suggesting that the origin of the parasites was unlikely to affect the cross‐specific infection susceptibility. Before the exposures, the snails were transferred to room temperature for 4 h, after which average cercarial density in the combined solution of all snails was estimated using 10 1 mL aliquots.

The fish were exposed to the eye parasite in 16 containers, each with 60 L of aerated lake water (+17.1°C) and 60 fish evenly from all crossing groups. Eight of the containers received a small amount of lake water with 18,000 infective cercariae (300 per fish) while the other eight received lake water without cercariae. Exposure time was 30 min after which the fish were transferred back to their rearing tanks. Note that *D. pseudospathaceum* does not transmit directly between fish which allowed maintenance of exposed and unexposed fish in the same tanks.

### 2.3 Predation Experiments

**FIGURE 1:**
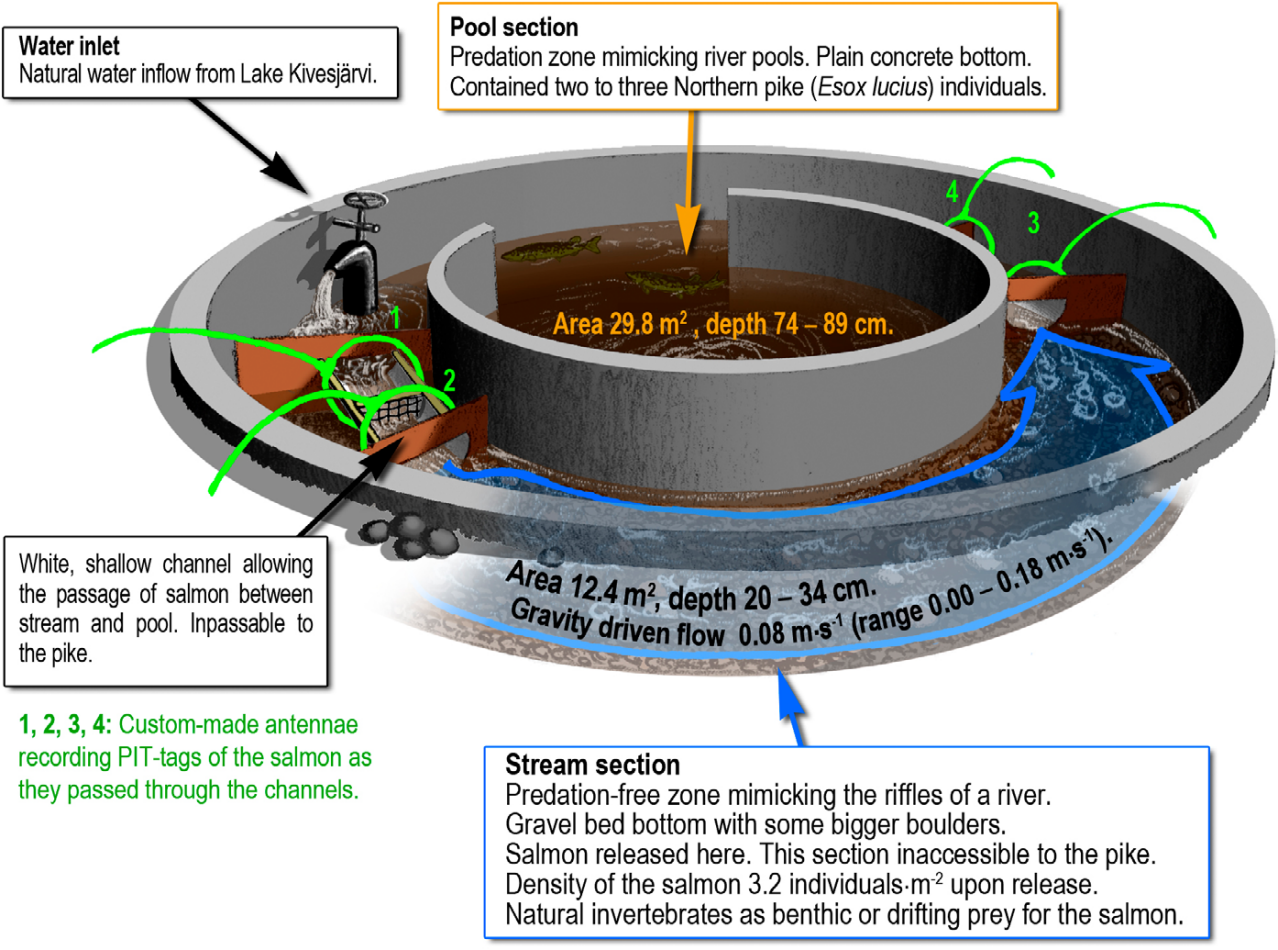
A schematic representation of the experimental predation tanks. The control tanks were otherwise identical to the predation tanks, but they lacked the predatory Northern pike in the central section mimicking a river pool. Drawing by: Aslak Eronen.

The first predation experiment was conducted from November 2020 to April 2021 with 1.5‐year‐old salmon (winter experiment) and the other experiment from May to June 2021 with 2‐year‐old salmon (summer experiment). The eight circular outdoor tanks used in the experiments were divided to two parts: a shallow, gravel‐bottomed outer ring mimicked stream section of a river and a deeper section with slower water flow mimicked pool area of a river (Figure 1). The sections were separated by two shallow flow‐through plastic channels equipped with custom‐made PIT‐antennae, recording movements of the individuals between the stream and pool sections (see Vainikka et al. 2012 for a description of the antenna system). Four of the eight tanks included pike in the pool section as predators. The pike had originally been caught from the wild and were maintained at the Kainuu Fisheries Research Station. Pike were prevented from entering the stream section with a net (mesh size 50 mm) placed in the middle of the antenna channels, but the net allowed salmon to pass between stream and pool sections. The other four tanks acted as control environments with no predators.

For both experiments (winter and summer), 320 fish were sampled at random from their rearing tanks, scanning the PIT‐tags of all caught individuals to find 80 salmon per crossing group, half of them infected with *D. pseudospathaceum* and another half uninfected. Thus, each of the eight experimental tanks contained ten salmon per crossing group, five infected and five uninfected (5 individuals × 2 infection statuses × 4 crossing groups × 8 replicate groups = 320 individuals in total; Appendix S1.1). All the fish were anesthetized and measured for body length and mass. For the individuals infected with *D. pseudospathaceum*, coverage of the parasite‐induced cataracts was determined for both eyes using a slit‐lamp microscope (Kowa SL‐15) (Karvonen and Seppälä 2008). Similarly, the absence of parasites within the eyes of uninfected fish was verified. All cataract measurements were conducted blind with respect to the crossing group and treatment.

For the winter experiment, the salmon were sampled and measured on 26–27th October 2020. Before the experiments were started, there were gates preventing the movement of salmon from the stream section to the pool section. The winter experiment was started by removing these gates on 3rd November after the fish had been acclimatizing for 6 days in the stream sections. In the winter experiment, each of the four predator tanks contained two pike (mean body weight 2.2 kg, range 1.6–3.0 kg, *n* = 8 pike). The winter experiment lasted for 154 days until the surviving salmon were collected and measured again on 6th April 2021. Water temperature during this period ranged from +1.1°C to +4.3°C (mean + 1.8°C), oxygen content from 8.5 mg L^−1^ to 12.4 mg L^−1^ (mean 10.6 mg L^−1^), and day length from 4 to 14 h. To verify the assumption that the lost individuals in the predation tanks had indeed died of predation, the bottom of the pool and stream sections were scanned after both experiments using a portable PIT‐reader. After the winter experiment, 69% of the tags of the lost individuals were recovered (35 out of 51), all in the pool sections. This suggests that these tags most likely were excreted by the pike after they had depredated the lost salmon.

For the summer experiment, the fish were measured 26th May and the gates preventing movement between stream and pool sections were removed after 4 days of acclimatization, on 30th May. In the summer experiment, each of the four predation tanks contained three pike (mean body weight 2.0 kg, range 1.2–3.0 kg, *n* = 12 pike). To minimize differences in risk of predation between the experiments (winter and summer), we used a higher number of pike per tank in the shorter summer experiment. The summer experiment was terminated after mortality was estimated, based on the PIT‐telemetry data, to be approximately equal to the winter experiment (i.e., after 28 days). Water temperatures during this period ranged from +7.8°C to +16.0°C (mean + 11.9°C), oxygen content from 6.9 mg L^−1^ to 10.1 mg L^−1^ (mean 8.7 mg L^−1^) and day length from 20 to 21 h. In the summer experiment, we found 47% of the tags of the lost individuals in the pool section (24 individuals out of 52) and 8% in the stream section (four individuals); cause of death for these four individuals was thus unknown.

### 2.4 Statistical Analyses

All statistical analyses were performed using R, version 4.2.1 (R Core Team 2022) and the R studio interface. Preliminary analyses indicated that the two hybrid groups (*LS × AS* and *AS × LS*) were intermediate to the purebred strains and did not differ from each other in body size or mortality (Appendix S1). They were, therefore, pooled into one hybrid group for the subsequent analyses.

#### 2.4.1 Predation‐Induced Mortality

Analysis of predation‐induced mortality (individuals that disappeared in the pool section) used only data from the tanks with predators. The data were analysed at two stages using generalized linear mixed models with a binomial error distribution from the R package glmmTMB (Brooks et al. 2017). First, we modelled the effect of the infection status on predation‐induced mortality (1 = infected, 0 = uninfected; Table 1a), after which the mortality was analysed separately for uninfected (Table 1b) and infected salmon (Table 1c). Lost individuals detected last in the stream section or not detected by the PIT‐antennae at all were excluded as they may have disappeared due to other reasons than predation. Model factors and variance structures were chosen based on Akaike information criteria (AIC‐values) starting from the most complex models with all interactions and excluding interactions and factors one by one (for candidate models and the detailed model selection processes, see Appendices S2–S4). Final model fits were evaluated using simulated residuals obtained from the package DHARMa (Hartig 2022). Paired post hoc comparisons were based on Šidák corrected values from the package emmeans (Lenth 2022).

**TABLE 1:** Results of the generalized linear mixed models for the probability of death due to piscine predation, based on the combined results from the winter and summer experiments (see also Figure 2). The response variable in the models is the binomial outcome of the experiment (survived/depredated). The model in (a) predicts mortality of all individuals within the predation tanks involving the factorial crossing group (*anadromous, hybrid, landlocked*), infection treatment (*uninfected/infected*), their interaction and experiment (*winter/summer*) as explanatory factors. The model in (b) only predicts mortality for the uninfected individuals involving crossing group as the sole explanatory factor. The model in (c) only predicts mortality for individuals infected with *D. pseudospathaceum* and includes crossing group as an explanatory factor and infection induced cataract coverage of the eye lenses as a continuous covariate. See also Figure 3.

	Wald *χ* ^2^	df	*p* > *χ* ^2^
*(a) Model on the effect of infection status, n = 319 individuals*			
Crossing group	5.29	2	0.071
Infection treatment	10.2	1	0.001
Crossing group × Infection treatment	12.37	2	0.002
Experiment	0.04	1	0.843
*(b) Model on uninfected salmon (infection controls), n = 159 individuals*			
Crossing group	8.46	2	0.015
*(c) Model on infected salmon, n = 160 individuals*			
Crossing group	2.83	2	0.243
Cataract coverage	22.57	1	< 0.001

#### 2.4.2 Body Size, Cataract Coverage, and the Effect of Predation on Overwintering Growth

Analysis of variance with Tukey‐adjusted post hoc comparisons were used to test possible differences in mean body size and condition factors (Ricker 1975) among the three crossing groups and two infection groups (Appendix S1.1). Further, mean cataract coverage was compared among the infected crossing groups. Similarly, for the surviving individuals of the 154‐day winter experiment, body sizes before and after the experiment were compared taking into account the predation treatment (Appendix S1). This was done to gain insights into the effect of the crossing group, predation, and infection on growth.

#### 2.4.3 Behavioural Analyses

The behavioural analysis only used data from salmon that survived until the end of the experiments. In the winter experiment, one predation‐free tank had to be excluded from the analyses due to technical issues that allowed the fish to enter the pool section before the experiment started. The behavioural data of the winter experiment thus comprised the movements of 228 individuals over the course of 154 days. The corresponding number in the summer trial was 269 fish over the course of 28 days.

The raw PIT‐data were configured into temporal resolution of 1 min using the TIRIS data‐logger program (Citius Solution, www.pitdata.net). The following variables were calculated from the data for each individual: (1) the proportion of time spent in the pool section indicating habitat use, (2) the latency time to enter the pool section indicating boldness (with shorter latency time indicating higher boldness), (3) the total distance moved indicating overall activity or explorative behaviour, (4) the number of changes between stream and pool sections also indicating overall activity or explorative behaviour, (5) the number of days on which the individual was recorded by the PIT‐antennae, another measure of activity, and (6) the median clock hour of the day an individual was moving, indicating circadian activity. All six behavioural variables were scaled between 0 and 1 (minimum and maximum values, respectively). Further, we log‐transformed (log_10_ *x* + 1) variables (3) (half rounds) and (4) (changes) due to right‐skewed distributions.

Next, these six behavioural variables were reduced to two uncorrelated principal components (PC) using principal component analysis (PCA) from the R package vegan (Oksanen et al. 2022). To improve the interpretability of the PCs, orthogonal varimax rotation was applied. After extracting the PC scores for each fish, we analysed the two PCs in linear mixed effects models with restricted maximum likelihood estimation method using the R package nlme (Pinheiro et al. 2022). The final models with appropriate variance structures were chosen based on AIC‐values and log‐likelihood‐tests from the package lmtest (Zeileis and Hothorn 2002). Model fits were graphically evaluated using residual plots. Winter and summer trials were analysed separately because of their different duration and different environmental conditions of the two seasons.

### 2.5 Statement on Animal Subjects

All animal experimentation was performed with permission obtained from the Finnish national Project Authorisation Board (formerly the Animal Experiment Board; license numbers: ESAVI/5184/04.10.07/2017 and ESAVI/8397/2021), and it follows the ABS/ASAB guidelines for ethical treatment of animals and complies with the current Finnish legislation. The salmon in the predation treatment had a safe zone in the stream section, which was inaccessible to the piscivorous pikes. Wellbeing of the pikes was ensured by regular feeding with dead roach ( *Rutilus rutilus* Linnaeus 1758) and smelt ( *Osmerus eperlanus* Linnaeus 1758). All salmon infected with *D. pseudospathaceum* were euthanized after the experiments with an overdosage of benzocaine.

## 3 Results

### 3.1 Predation‐Induced Mortality

In the winter experiment, overall mortality was 1.8% (three individuals) in control tanks and 30.8% (49 individuals) in predation tanks. In the summer experiment, overall mortality was 0.6% (one individual) in control tanks and 31.3% (50 individuals) in predation tanks. Predation‐induced mortality was 28.8% across the experiments (46 individuals in both experiments), excluding seven individuals that died of unknown causes.

**FIGURE 2:**
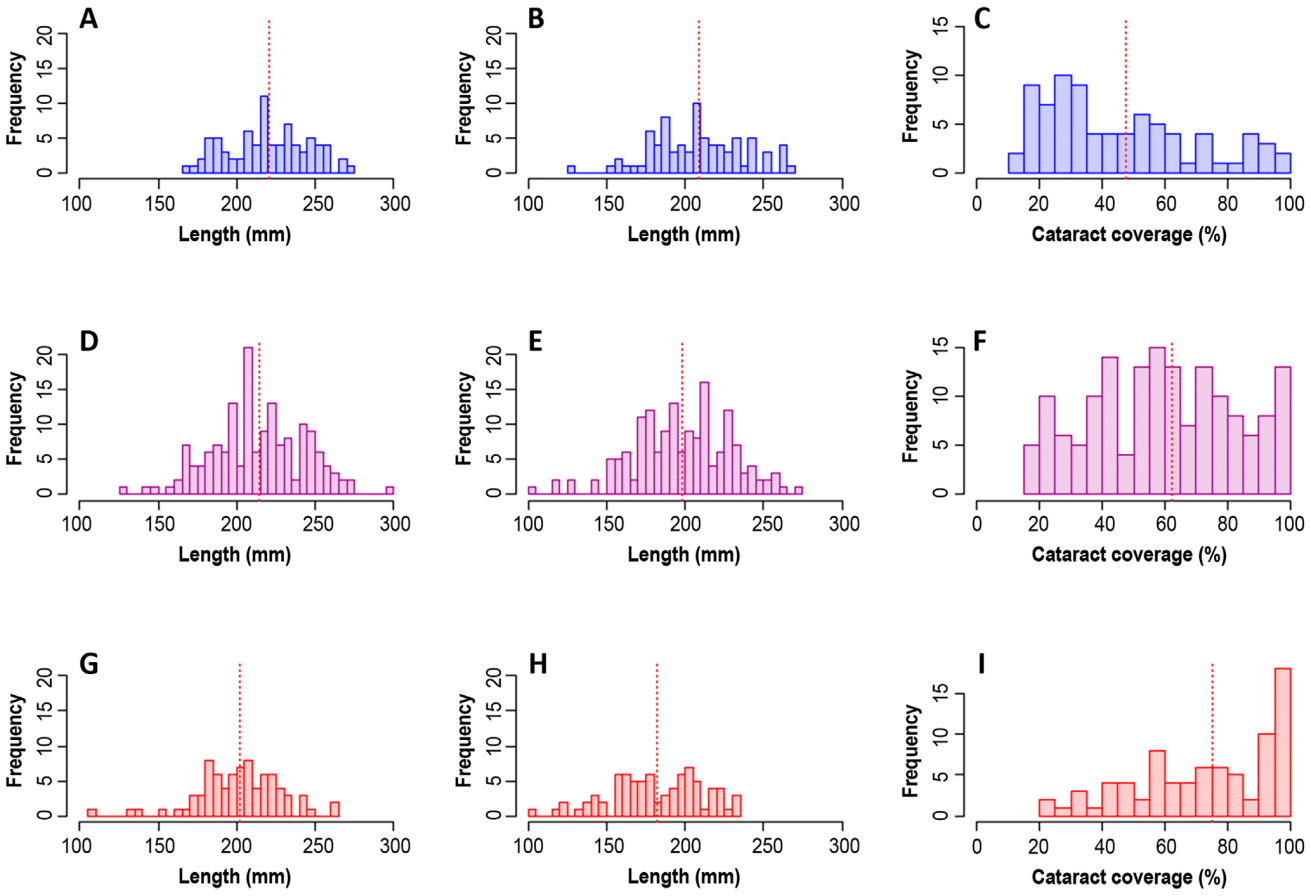
Histograms of the lengths of uninfected landlocked salmon (A), landlocked salmon infected with *D. pseudospathaceum* (B), cataract coverages of infected landlocked salmon (C), lengths of uninfected hybrid salmon (D), lengths of infected hybrid salmon (E), cataract coverages of infected hybrid salmon (F), lengths of uninfected anadromous salmon (G), lengths of infected anadromous salmon (H) and cataract coverages of infected anadromous salmon (I). This data contains all 640 salmon involved in both the winter and the summer experiment. Means per group are indicated by vertical red dotted lines.

Predation‐induced mortality was significantly higher among the infected salmon (mean ± S.E. = 37.4% ± 8.4%) than among the uninfected salmon (16.8% ± 5.4%; *p <* 0.001 for a pairwise comparison; Table 1a; Figure 2A). Although the effect of the crossing group on predation‐induced mortality was only marginally significant, there was a statistically significant interaction between the infection status and crossing group (Table 1a). Post hoc comparisons revealed that infected anadromous salmon had higher predation‐induced mortality (58.8% ± 11.0%) than either uninfected anadromous salmon (9.9% ± 5.4%) or uninfected hybrid salmon (13.4% ± 5.2%) (Figure 2A). Further, infected hybrid salmon showed marginally lower mortality (28.6 ± 8.1%) than infected anadromous salmon (58.8% ± 11.0%; *p =* 0.056; Figure 2A). Interestingly, neither infected (27.2% ± 9.5%) nor uninfected landlocked salmon (32.7% ± 10.3%) differed significantly from the other groups in predation‐induced mortality.

When only the uninfected salmon were analysed, the final model indicated a significant difference among the crossing groups (Table 1b; Appendix S3): uninfected landlocked salmon showed higher predation‐induced mortality (37.2% ± 9.4%) than uninfected hybrid (14.5% ± 4.8%) or anadromous salmon (10.6% ± 5.4%; Figure 2B).

For the infected salmon, the final model indicated that eye cataract coverage was positively associated with predation‐induced mortality (Figure 2C; Appendix S4), while the independent effect of the crossing group was insignificant (Table 1c). Consequently, the mean cataract coverage of infected individuals that survived from predation was 51.7 ± 22.2 (% ± S.D.), while it was 78.3 ± 19.5 among the individuals that were depredated (mean across both experiments).

### 3.2 Body Size, Cataract Coverage, and the Effect of Predation on Overwintering Growth

**FIGURE 3:**
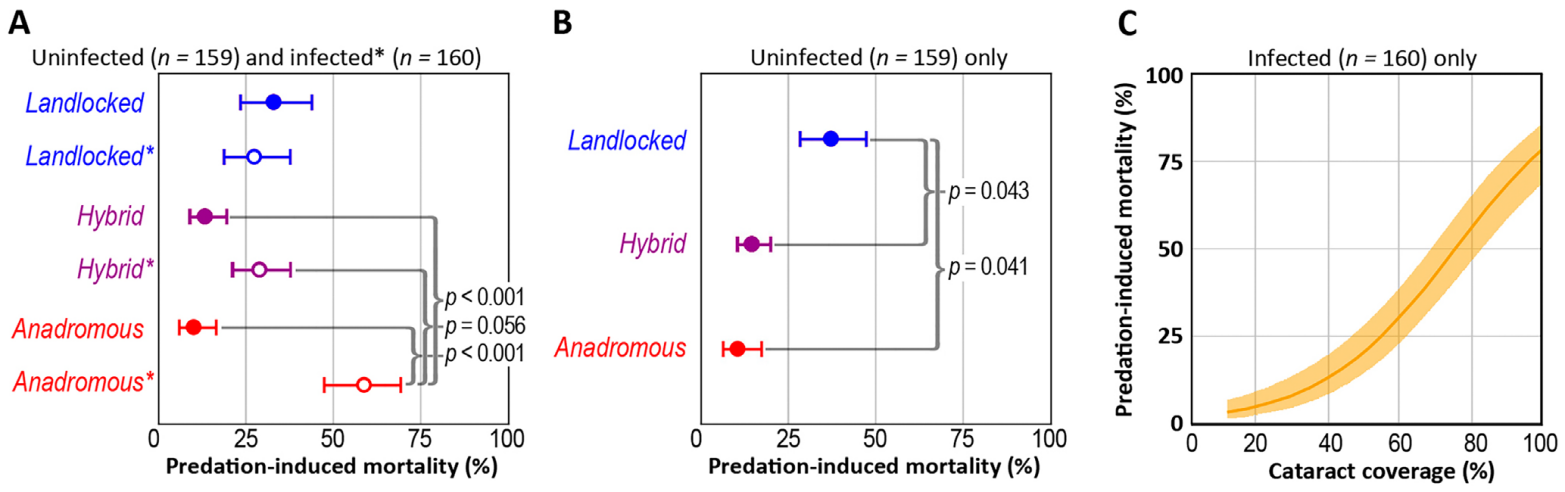
The effects of hybridization and infection with *D. pseudospathaceum* on juvenile salmon mortality induced by piscine predation, based on the combined results from winter and summer experiments. Predicted predation‐induced mortality is based on the data collected from the predation tanks only, excluding four individuals that died of unknown causes. Graph (A) shows the predicted predation‐induced mortality per crossing group accounting for the binomial infection status (uninfected/infected*), with the whiskers representing standard errors. The curled brackets show statistically significant pairwise comparisons with corresponding *p*‐values. Graph (B) shows predicted predation‐induced mortality per crossing group (±S.E.) among uninfected (infection controls) fish only. Graph (C) shows the predicted probability of predation‐induced mortality plotted against the coverage of parasite‐induced cataracts in the eye lenses of infected salmon (logistic regression curve ± S.E. shaded). See also Table 1.

The analyses on body size showed that the infected salmon were significantly smaller than the uninfected salmon at the beginning of the experiments (Figure 3). Further, in both experiments, anadromous salmon were on average smaller than landlocked salmon or hybrids, regardless of infection status (Figure 3; more details in Appendix S1). In both experiments, parasite‐induced cataract coverage varied similarly among the crossing groups: landlocked salmon showed a lower cataract coverage than the anadromous salmon, with hybrids being intermediate (*p <* 0.039 for all pairwise comparisons, except *LS × AS* vs. *AS × LS*: *p =* 0.259) (Figure 3; Appendix S1). Further, cataract coverage correlated negatively with body size before the experiments (Figure S1.1). Before the winter experiment, cataract coverage also correlated negatively with the condition index, where a cataract coverage of 80% seemed to be a threshold value, after which the condition index dropped notably (Figure S1.2).

A total of 268 individuals (83.8%) survived the winter experiment and were measured again on 6th April 2021 (Table S1.6). Salmon subjected to predation had lost, on average, 11.1% ± 4.5% (±S.D.) of their body mass while the salmon in control tanks had instead increased their body mass by 9.3% ± 11.3% (*p <* 0.001). Specifically, *LS × LS* and *AS × LS* had gained more weight (15.5% ± 9.3% and 10.4% ± 11.8%, respectively) in the predator‐free control treatment than *AS × AS* (3.5% ± 9.6%), while weight gain in *LS × AS* (7.9% ± 11.1%) was intermediate to the other groups. Similarly, the mean condition index of the salmon in the predation treatment decreased by 4.4% ± 4.4%, while it increased by 1.7% ± 5.0% in the control treatment (*p <* 0.001; Appendix S1). Within the predation treatment, cataract coverage had no effect on growth, that is, the uninfected salmon had lost weight similarly to the infected salmon. In the control treatment, in contrast, the individuals with the most severe cataracts were able to grow and improve their condition even more than the uninfected individuals (Figure S1.2).

### 3.3 Behaviour

**FIGURE 4:**
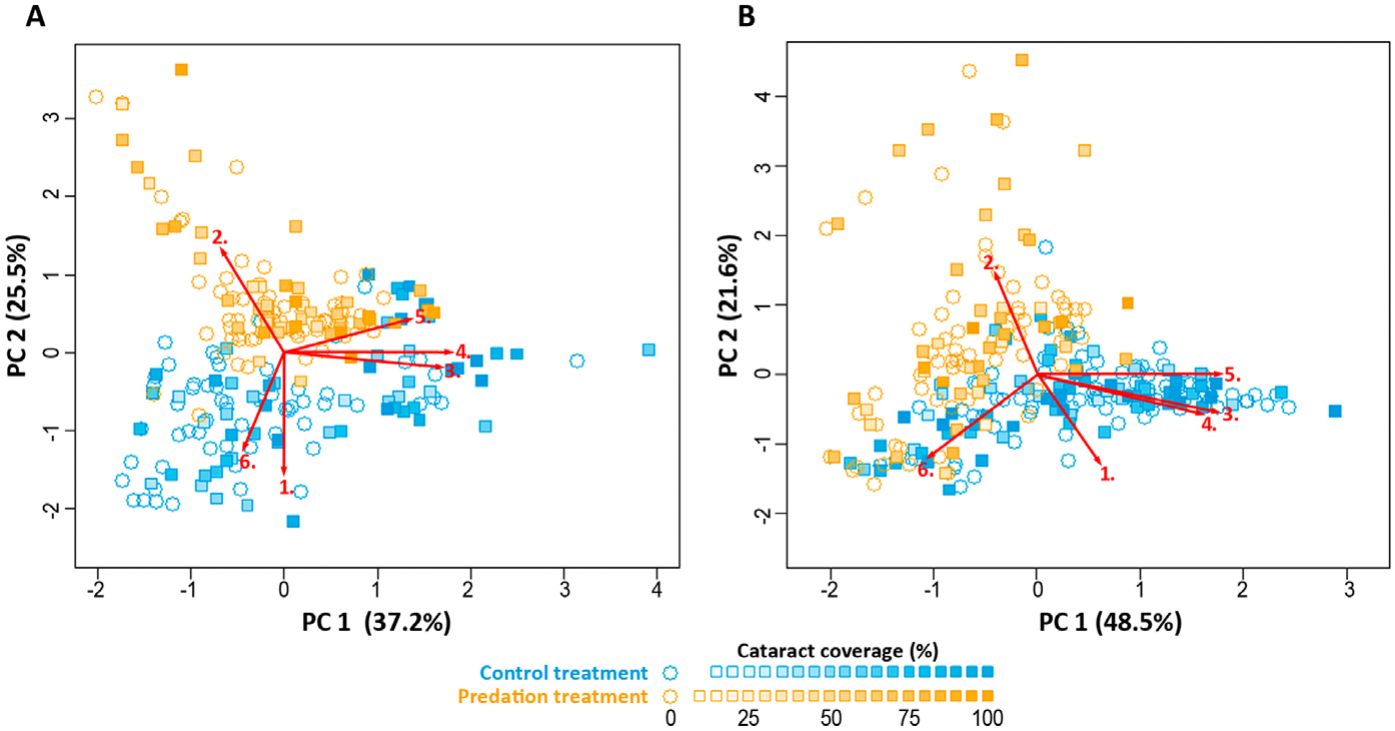
Graphical representations of the first two varimax‐rotated principal component axes extracted from six behavioural variables derived from PIT‐telemetry of the salmon in the winter experiment (A) and in the summer experiment (B). Yellow points represent individuals in the predation treatment and blue points represent individuals in the predation‐free control treatment. Uninfected individuals are shown as empty circles and infected individuals as squares, with the shade of the square fills representing cataract coverage of the eye lens (see legend for colour scale). The red arrows represent the varimax‐rotated loadings of the six behavioural variables in a two‐dimensional space: 1 = the proportion of time spent in the pool section of the tanks, 2 = the latency time before entering the pool‐section for the first time, 3 = the log‐transformed total distance swam during the experiment, 4 = the log‐transformed number of changes between stream and pool sections, 5 = the number of days when an individual was recorded moving, and 6 = the median clock hour of the day an individual was recorded moving, i.e. circadian activity.

In both experiments, the PCA resulted in two principal components with eigenvalues higher than 1 (Figure 4A,B). In the winter experiment, PC 1 explained 37.2% and PC 2 explained 25.5% of the total variation in the original behavioural traits. In the summer experiment, PC 1 explained 48.5% and PC 2 explained 21.6% of the total behavioural variation. PC 1 correlated positively with the three variables reflecting the movement activity of the salmon (variables 3, 4 and 5 in Figure 4) in both experiments (see Appendix S5 for more details). PC 2, instead, reflected habitat use and circadian activity (variables 1, 2 and 6 in Figure 3).

**FIGURE 5:**
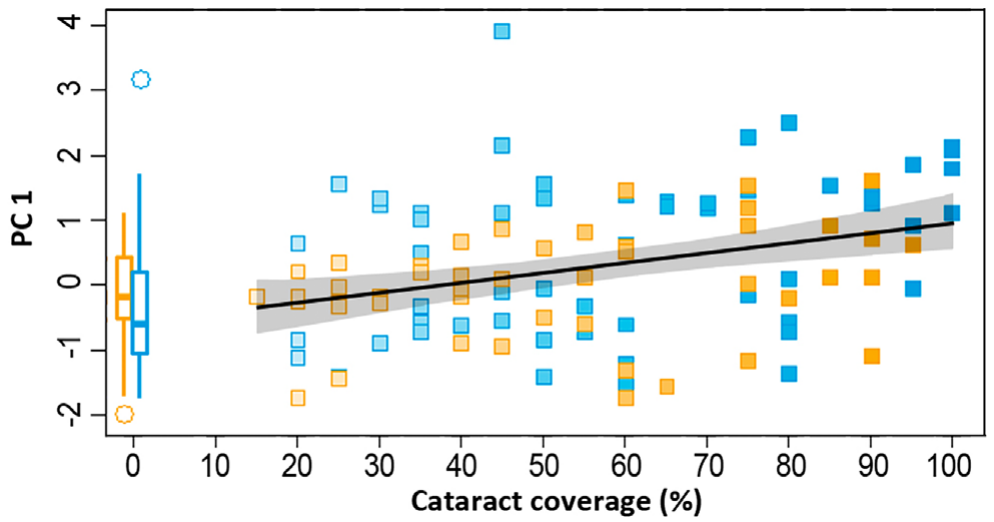
The first varimax‐rotated principal axis of the winter experiment plotted against cataract coverage. The first principal component was extracted from six behavioural variables derived from PIT‐telemetry and was interpreted to mostly reflect overall activity: The higher the value on PC 1, the higher the activity level. The boxes on the left‐hand side of the graph represent the median PC 1 value of the uninfected salmon per treatment (yellow = predation, blue = control) and their interquartile ranges (25th and 75th percentiles), while the whiskers represent 1.5 times the interquartile range. Similarly, for the individuals infected with D. pseudospathaceum, yellow squares represent individuals subjected to predation treatment and blue squares represent individuals subjected to predation‐free control treatment. The black line shows a spline among the infected salmon where the shaded areas give the 0.95 confidence intervals (see also Table 2a).

The linear mixed model on PC 1 (correlating positively with activity) in the winter experiment included only cataract coverage as a highly significant continuous explaining variable (Table 2a; Figure 5). Hence, activity of the salmon in winter was mainly determined by the severity of the infection with *D. pseudospathaceum*: the higher the cataract coverage, the more active the salmon were, regardless of predation (Figure 5). The final linear mixed model on PC 2 (correlating with habitat use and circadian activity) in the winter experiment, instead, included predation treatment (predation vs. control), cataract coverage, and their interaction as explanatory variables (Table 2b). In addition to cataract coverage, also predation thus clearly affected fish behaviour during overwintering: if there were predators present on the pool‐side, salmon were more hesitant to enter the pool section and spent most of their time in the stream section of the tanks.

**TABLE 2:** Results of the linear mixed effects model on behaviour, as inferred from PIT‐telemetry of the winter predation experiment. The principal components were extracted from six behavioural variables derived from PIT‐telemetry. PC 1 (a) was interpreted to correlate positively with activity levels, while PC 2 (b) reflected habitat use and circadian activity patterns.

	df_num_	df_den_	*F*	Effect size (*η* ^2^)	*p*
*(a) Winter experiment, varimax‐rotated PC 1*					
Intercept	1	220	< 0.001	< 0.01	0.982
Cataract coverage	1	220	24.344	0.10	< 0.001
*(b) Winter experiment, varimax‐rotated PC 2*					
Intercept	1	219	0.286	< 0.01	0.594
Predation	1	5	54.285	0.92	0.001
Cataract coverage	1	219	5.462	0.02	0.020
Predation × Cataract coverage	1	219	6.614	0.03	0.011

**FIGURE 6:**
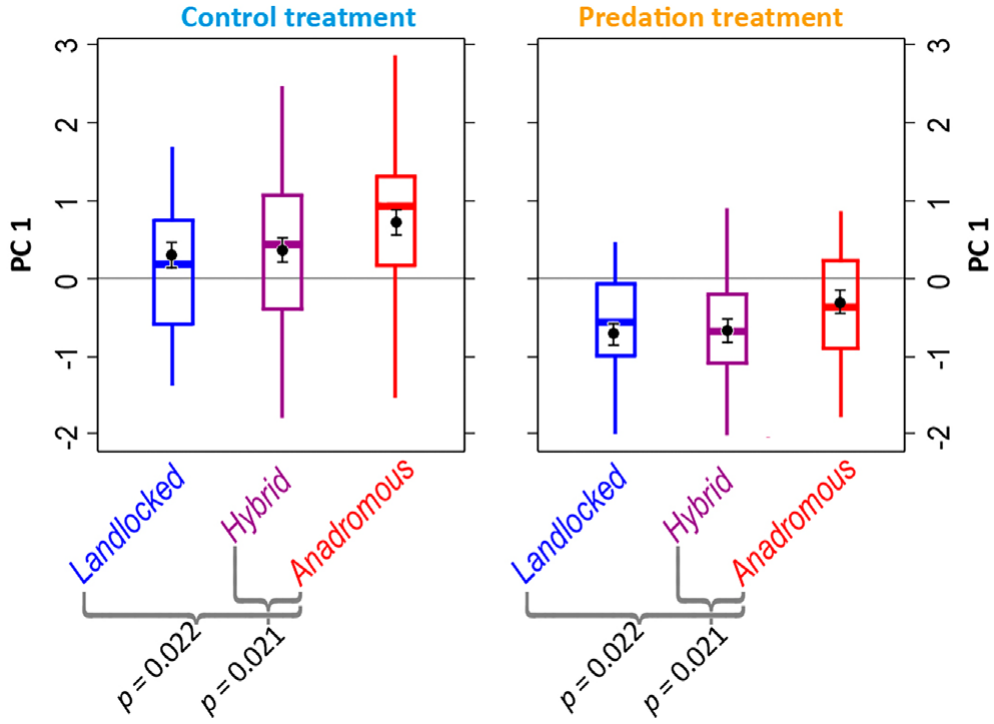
Box plots of the first principal component axis of the summer experiment plotted against salmon crossing groups within predation‐free control and predation treatments. The PC was extracted from six behavioural variables derived from PIT‐telemetry and was interpreted to mostly reflect overall activity: The higher the value on PC 1, the higher was the activity level of salmon. The boxes represent the medians and their interquartile ranges (25th and 75th percentiles), and the whiskers represent 1.5 times the interquartile range. Modelled means are given as black dots, with the whiskers representing standard errors (Table 3a). The curled brackets give statistically significant pairwise comparisons (with corresponding p‐values) among the crossing groups within each treatment.

In the summer experiment, the linear mixed model of PC 1 included predation treatment (predation vs. control) and crossing group as explanatory factors, both significant (Table 3a; Figure 6). In the summer experiment, the salmon were, therefore, significantly more active in the absence of predators than if predators were present (Table 3a; Figure 6). Further, irrespective of the predation treatment, anadromous salmon obtained higher scores on PC 1 than landlocked salmon or hybrids, thus being more active, on average, than salmon in the latter two groups (Figure 6). The final linear mixed model on PC 2 only included predation treatment as a marginally significant explanatory factor (Table 3b).

**TABLE 3:** Results of the linear mixed effects model on behaviour, as inferred from PIT‐telemetry of the summer predation experiment. The principal components were extracted from six behavioural variables derived from PIT‐telemetry. PC 1 (a) was interpreted to correlate positively with activity levels, whereas PC 2 (b) reflected habitat use and circadian activity patterns.

	df_num_	df_den_	*F*	Effect size (*η* ^2^)	*p*
*(a) Summer experiment, varimax‐rotated PC 1*					
Intercept	1	259	1.36	< 0.01	0.245
Predation	1	6	30.572	0.84	0.002
Crossing group	1	259	6.507	0.05	0.002
*(b) Summer experiment, varimax‐rotated PC 2*					
Intercept	1	261	0.151	< 0.01	0.698
Predation	1	6	5.496	0.48	0.058

## 4 Discussion

Following the genetic rescue theory, controlled supplementation of inbred salmonid populations with small numbers of hybrid individuals to prevent complete extinction has been increasingly acknowledged (Burridge 2019; Kovach et al. 2022; Pregler et al. 2023; White, Rash, and Kazyak 2023). Up to date, however, genetic rescue has been used rarely in conservation biology mainly because of concerns of outbreeding depression (Whiteley et al. 2015; Ralls et al. 2018; Bell et al. 2019; Chan, Hoffmann, and van Oppen 2019). Further, the important role of pathogens and parasites is often neglected in genetic rescue studies (but see Malmberg et al. 2019; Klemme et al. 2021, 2024; Lewis et al. 2023). Here, we evaluated fitness effects of hybridization by crossbreeding the critically endangered landlocked salmon with one of its closest living relatives, anadromous Baltic salmon. We found that hybridization improved survival from piscine predation relative to the landlocked salmon, with predation‐induced mortality of uninfected landlocked salmon being 37% compared to 15% among the uninfected hybrids. In contrast, predation‐induced mortality of hybrids infected with *D. pseudospathaceum* (29%) was comparable to that of infected landlocked salmon (27%). This suggests that the infection with this naturally prevalent parasitic eye fluke removed the beneficial effect of hybridization. A previous study using the same crossing groups at an earlier life history stage (age 1+) found a predation‐induced mortality of 41% among uninfected landlocked salmon and 44% among uninfected hybrid salmon (corresponding numbers were 50% and 65% in infected fish) (Klemme et al. 2024). This implies that the survival advantage of hybridization, particularly under predation risk, can be context‐ or age‐specific.

Consistent with our hypothesis and the results of previous studies (Karvonen et al. 2023; Klemme et al. 2024), the eye infection by the parasitic fluke *D. pseudospathaceum* increased the susceptibility of juvenile salmon to piscine predation from 17% to 37% (corresponding numbers were 45% and 63% in Klemme et al. 2024). As also expected, the infection had the most severe impact on survival among the anadromous salmon, which has lower resistance to the parasite compared to landlocked salmon (Klemme et al. 2021, 2024). Further, we found that cataract coverage correlated negatively with predation survival and growth among all crossing groups (see also Karvonen et al. 2023; Klemme et al. 2021, 2024). Overall, these results suggest that the significantly higher cataract coverage observed in anadromous salmon mostly explained the higher mortality among the infected anadromous salmon (59%). As the possible positive effects of hybridization can be removed by the infection, the net fitness outcome of hybridization in natural settings may turn negative (Eronen et al. 2023; Klemme et al. 2021, 2024). Even though the parasite can be prevalent in landlocked salmon smolts intended for stocking (Kuukka, Peuhkuri, and Kolari 2006; Seppänen et al. 2008), its abundance in the populations natural habitat is yet unknown.

In addition to the negative effect of *D. pseudospathaceum* on salmon survival and size, we also found that the coverage of parasite‐induced cataracts correlated positively with the overall activity of the salmon during overwintering. It is possible that infected individuals needed to compensate the reduced foraging success caused by the cataracts by foraging more actively (see also Crowden and Broom 1980). Reduced foraging efficiency of infected fish was also supported by the negative correlation between cataract coverage and body size, previously reported for example in European whitefish ( *Coregonus lavaretus* Linnaeus 1758; Karvonen and Seppälä 2008), rainbow trout ( *Oncorhynchus mykiss* Waldbaum 1792; Kuukka‐Anttila et al. 2010), and Atlantic salmon (Karvonen et al. 2023). Together with the reduced vision, higher movement activity could also partly explain the higher susceptibility of the infected salmon to piscine predation, at least in the winter experiment. However, we did not observe differences in overwintering activity among the crossing groups. This contrasts with the results of an earlier study with the same crossing groups, where landlocked salmon (underyearlings) were consistently more active than anadromous salmon with hybrids being intermediate (Eronen et al. 2023). Thus, the behavioural data of the present study should be interpreted with caution in explaining the observed survival differences.

In the summer experiment, however, activity was influenced by both crossing group and predation but not by the infection. In summer, the presence of predators reduced the overall activity levels, with the fish in predation tanks showing lower activity compared to control tanks. Further, we found that the anadromous salmon were, on average, more active compared to landlocked salmon and hybrids (see also Klemme et al. 2024 for similar results). For unknown reasons, however, we did not detect clear signs of smolt migration during the summer experiment, even though some of the salmon had undergone smoltification‐related phenotypic changes (e.g., change in coloration) and temperatures during the experiment were optimal for the onset of smolt migration. Nevertheless, we can conclude that more active behaviour combined with a higher eye cataract coverage probably exposed infected anadromous salmon to higher predation risk during summer compared to landlocked and hybrid salmon.

In both experiments, the threat of predation made the salmon to prefer the shallow stream section of the experimental tanks. In absence of predation, on the contrary, salmon did not hesitate to enter the pool section with deeper and more stagnant water and spent more time there than in the stream section. This suggests that salmon were aware of the predators in the pool section, regardless of their infection status (see also Klemme et al. 2024; Karvonen et al. 2023). Overwintering juvenile salmonids are known to move to deeper, slow current parts of the rivers, whereas in summer, they prefer fast‐flowing stream sections (Egglishaw and Shackley 1977; Metcalfe and Thorpe 1992; Huusko et al. 2007). Even though the shift to winter habitats is likely to expose salmonids to higher piscine predation, it can also save energy and protect from starvation (Egglishaw and Shackley 1977; Metcalfe and Thorpe 1992; Parrish, Hawes, and Whalen 2004). This is supported by the present data, where salmon in the predation treatment, spending most of their time in the stream section of the tanks, had decreased in body weight and condition during overwintering (Figure S1.1). Meanwhile, salmon in the control tanks were able to grow and improve their condition in the predation free pool sections, regardless of their infection status. Minimal growth or reduction in size have also been reported in previous overwintering studies, where juvenile Atlantic salmon have been monitored in natural rivers (Egglishaw and Shackley 1977; Metcalfe and Thorpe 1992; Letcher, Gries, and Juanes 2002).

In summary, our results imply that hybridizing the landlocked salmon with genetically more diverse anadromous conspecifics can yield both positive and negative fitness effects, depending on parasitism. Among the uninfected fish, we found signs that hybridization could improve survival in relation to the landlocked salmon, the target of the potential genetic rescue actions. Ultimately the outcome of possible genetic rescue attempts, however, depends not only on the prevalence of predators during critical life‐history stages of juvenile salmon but also on the magnitude of parasite exposure they encounter. Further evaluation of the overall net effect of hybridization needs to be extended to the whole life cycle and to subsequent generations where the landlocked salmon would represent a majority of the backcrossed genome. Genetic recombination in such backcrosses could reveal outbreeding depression not visible in the first‐generation hybrids (Gharrett et al. 1999; Bell et al. 2019). On the other hand, some of the backcrosses could also show a combination of advantageous alleles from both parental populations (e.g., Poirier et al. 2019; Lewis et al. 2023, see also the review by Frankham 2016). In the present system, such benefits could include resistance to parasitism by *D. pseudospathaceum* combined with improved ability to evade predators. Captive maintenance of the hybrid population along with careful evaluation of the hybrid backcrosses is therefore crucial for possible future genetic rescue actions with the Lake Saimaa landlocked salmon.

A particular feature of the landlocked salmon compared to many other populations targeted with genetic rescue actions is that it is essentially a captive‐bred population. This means that the introduction of new alleles and the relative proportion of the foreign genome in the hybrids can be completely controlled by hatchery propagation. However, as long as the population is dependent on captive breeding, the challenge of hatchery selection (domestication) remains (Houde, Fraser, and Hutchings 2010; Milla et al. 2020). Consequently, genetic rescue alone is unlikely to be a sufficient conservation measure in saving the landlocked salmon population, which is why the primary objective should be strengthening of natural reproduction. Further, even if genetic rescue of the landlocked salmon were to be successful in the long‐term, implementation of such actions in the natural population would also mean that its genetic uniqueness would be lost. So the main question of preserving a genetically distinct landlocked salmon population even with the risk of extinction versus sacrificing the distinctiveness for viability remains to be evaluated in the future (see also Burridge 2019 for a similar situation).

## Conflicts of Interest

The authors declare no conflicts of interest.

## Supporting information


Appendix S1.

## Data Availability

All data for this study are archived online in the Dryad digital repository: https://doi.org/10.5061/dryad.c59zw3rhd.
