# Supplementary material for: The Effects of Hybridization and Parasite Infection on the Survival and Behaviour of Endangered Landlocked Salmon Subject to Predation—Implications for Genetic Rescue
Source: Evol Appl. 2024 Dec 13;17(12):e70056. doi: 10.1111/eva.70056 (PMC11645445; doi:10.1111/eva.70056)
Supplement: Supplementary file 1 — Appendix S1. [file EVA-17-e70056-s001.pdf]

Supporting information:

The effects of hybridization and parasite infection on predation survival and behaviour of endangered landlocked salmon – implications for genetic rescue

This file contains a table on fish body sizes, condition factors and cataract coverages before pooling the hybrid salmon, as well as analyses of variance conducted on the different measured attributes in the winter and summer experiments. Further, we provide plots visualizing the correlations between cataract coverage with body size and condition factor. We also provide additional information and analyses on the measurements conducted on the surviving fish of winter experiment fish after the experiment was terminated, on the 6th April 2021. The model selection processes of the mortality models are explained in detail in sections S2, S3 and S4. Further, we show plots of the activity patterns and likely predation events as deduced from the PIT-telemetry recorded during the two experiments. Finally, we plot the two principal PCA-axes of the winter and summer experiments against the six behavioral variables analyzed to help in the interpretation of the PCA.

Contents:

S1 Body sizes, condition factors and cataract coverages:
S1.1 Calculation of the condition factors....2
Table S1.1 Body sizes, condition factors and cataract coverages before the summer and winter experiment...2
Figure S1. 1 Body size and condition factor plotted against cataract coverage in winter and summer....3
Table S1. 2 Analyses of variance of the measurements conducted before the winter experiment....4
Table S1. 3 Pair-wise Post Hoc comparisons among the crossing groups before the winter experiment....4
Table S1. 4 Analyses of variance of the measurements conducted before the summer experiment....5
Table S1. 5 Pair-wise Post Hoc comparisons among the crossing groups before the summer experiment....5
Table S1. 6 Body sizes, condition factors and cataract coverages before and after the winter experiment....6
Table S1. 7 Analyses of variance of the changes in body size during the winter experiment....7
Table S1. 8 Pair-wise Post Hoc comparisons of the changes in body size among the crossing groups....7
Figure S1. 2 Condition factors plotted against cataract coverage before and after the winter experiment....8

S2 The model selection process leading to the mortality model in Table 2a: all salmon
Step 1 The candidate models....9
Step 2 Evaluating all candidate models against each other....10
Step 3 Choosing a variance structure for the final model....11
Step 4 Residual diagnostics of the final model....12

S3 The model selection process leading to the mortality model in Table 2b: uninfected salmon
Step 1 The candidate models....13
Step 2 Evaluating all candidate models against each other....14
Step 3 Choosing a variance structure for the final model....14
Step 4 Residual diagnostics of the final model....15

S4 The model selection process leading to the mortality model in Table 2c: infected salmon
Step 1 The candidate models....16
Step 2 Evaluating all candidate models against each other....17
Step 3 Choosing a variance structure for the final model....17
Step 4 Residual diagnostics of the final model....18

S5 The behavioural variables derived from PIT-telemetry recorded during the experiments:
Figure 1 Relative activity of the fish during the winter experiment, likely predation events....19
Figure 2 Relative activity of the fish during the summer experiment, likely predation events....20
Figure 3 Winter experiment: PC1 plotted against the six behavioral variables....21
Figure 4 Winter experiment: PC2 plotted against the six behavioral variables....22
Figure 5 Summer experiment: PC1 plotted against the six behavioral variables....23
Figure 6 Summer experiment: PC2 plotted against the six behavioral variables....24

Appendix S1 Body sizes, condition factors and cataract coverages:

S1.1 Calculation of the condition factors

Condition factors of all salmon involved in the experiments were calculated as:

$$Condition\ \text{factor} = \frac{Body\ weight\ (g)}{a \times Body\ length\ (cm)^b} \quad \text{(Ricker, 1975)}$$

That is, we divided the observed body weights by the expected body weights, which were calculated from the populations mean logarithmic relationship between body weight and body length, separately for the winter experiment fish and the summer experiment fish. In the winter experiment, *a* was 0.006295 and *b* was 3.173299 and in the summer experiment *a* was 0.006036 and *b* was 3.152179.

Ricker, W.E. (1975). Computation and Interpretation of Biological Statistics of Fish Populations. *Bulletin of the Fisheries Research Board of Canada, 191*(1), 1-382.

Table S1.1. The number of salmon individuals (n) per crossing group (*Female parent x male parent, LS = Landlocked salmon, AS = Anadromous salmon*) and infection treatment (0 = *uninfected*, 1 = *infected with D. pseudospathaceum*), their mean (± sd) body length (mm), mass (g), condition factor and parasite induced cataract coverage of the eye lenses (%). Table (a) indicates the measurements before the start of the winter experiment, 27th of October 2020 and table (b) gives the measurements at the start of the summer experiment 27th of May 2021. Note that the hybrids are not pooled in this table, but pooling was done after the statistical testing (see next pages).

(a) Winter experiment 2020/2021 with age 1.5 salmon

Crossing group	Infection status	n	Body length	Body mass	Condition factor	Cataract coverage	Survivors
LS x *LS*	0	20/20	202 ± 18	88.5 ± 21.8	1.00 ± 0.06	-	19/13
	1	20/20	190 ± 22	75.1 ± 25.7	1.00 ± 0.07	45 ± 23	19/12
LS x *AS*	0	20/20	199 ± 23	88.5 ± 28.8	1.02 ± 0.06	-	20/13
	1	20/20	178 ± 25	61.8 ± 25.7	0.98 ± 0.13	63 ± 22	20/14
AS x *LS*	0	21/19	194 ± 23	82.0 ± 28.1	1.03 ± 0.04	-	20/17
	1	20/20	179 ± 24	63.6 ± 24.5	1.01 ± 0.07	60 ± 26	20/15
AS x *AS*	0	20/20	187 ± 24	72.9 ± 21.8	1.03 ± 0.08	-	20/19
	1	20/20	163 ± 25	46.8 ± 22.4	0.96 ± 0.14	74 ± 23	20/7

(b) Summer experiment 2021 with age 2 salmon

Crossing group	Infection status	n	Body length	Body mass	Condition factor	Cataract coverage	Survivors
LS x *LS*	0	20/20	239 ± 17	140.0 ± 32.7	1.03 ± 0.05	-	19/12
	1	20/20	227 ± 23	121.0 ± 36.2	1.03 ± 0.06	50 ± 26	20/15
LS x *AS*	0	20/20	232 ± 25	124.0 ± 41.0	0.99 ± 0.06	-	20/19
	1	20/20	219 ± 20	104.0 ± 28.9	1.00 ± 0.06	68 ± 24	20/11
AS x *LS*	0	20/20	232 ± 24	127.0 ± 41.1	1.00 ± 0.06	-	20/17
	1	20/20	218 ± 28	106.0 ± 42.2	1.01 ± 0.07	57 ± 21	20/11
AS x *AS*	0	20/20	217 ± 21	98.5 ± 29.8	0.98 ± 0.07	-	20/16
	1	20/20	200 ± 22	78.0 ± 24.9	0.99 ± 0.07	77 ± 22	20/9

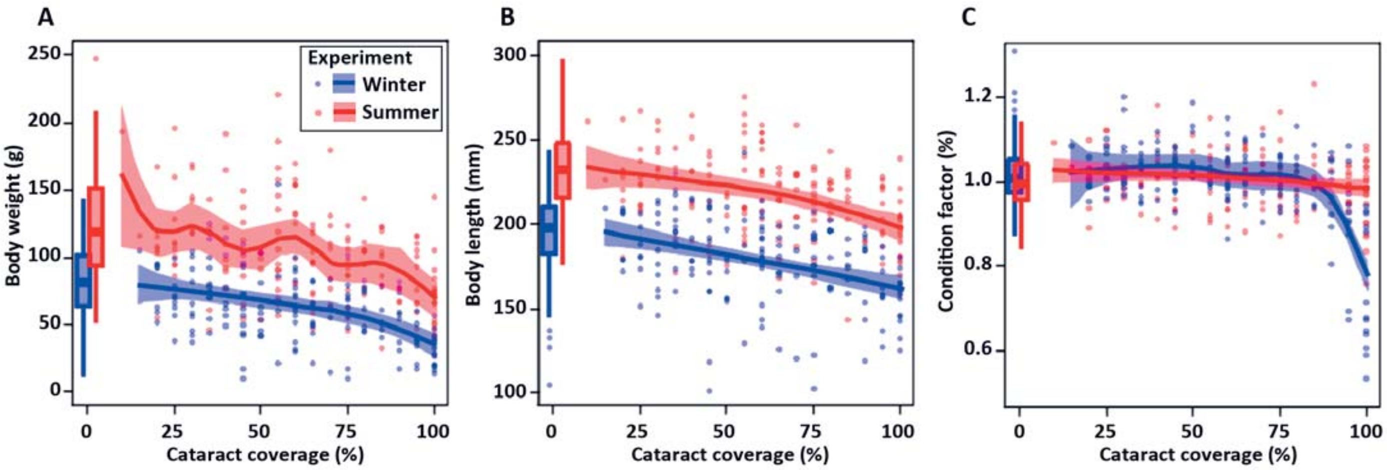

Figure S1.1. Body mass (a), body length (b) and condition factor (c) plotted against cataract coverage of all salmon involved in the two experiments. Blue color stands for the winter experiment (measured 27th of October 2020) and red color for the summer experiment (measured 26th May 2021). The boxplots for uninfected fish (cataract coverage 0 %) represent the medians and their interquartile ranges (25th and 75th percentiles), with the whiskers representing 1.5 times the interquartile range. For the infected fish the means are represented by loess smoothed splines where the shaded areas give the 0.95 confidence intervals.

Table S1.2. Results of the analysis of variance conducted on salmon body masses (a), body length (b), condition factor (c) and parasite induced cataract coverage of the eye lenses (d) before the winter experiment, as measured on the 27th of October 2020. For paiwise comparisons among crossing groups in (a), (b) and (d), see table S1.3 below.

**(a) Body mass**	Df	F	P
Crossing group	3	10.92	**< 0.001**
Infection treatment	1	57.46	**< 0.001**
Crossing group × Infection treatment	3	1.28	0.276
**(b) Body length**	Df	F	P
Crossing group	3	11.34	**< 0.001**
Infection treatment	1	48.72	**< 0.001**
Crossing group × Infection treatment	3	1.16	0.325
**(c) Condition factor**	Df	F	P
Crossing group	3	1.26	0.286
Infection treatment	1	8.58	**0.004**
Crossing group × Infection treatment	3	2.40	0.068
**(d) Cataract coverage**	Df	F	P
Crossing group	3	10.256	**< 0.001**

Table S1.3. P-values of Tukey-adjusted pairwise Post Hoc comparisons among crossing groups (*Female parent x male parent, LS = Landlocked salmon, AS = Anadromous salmon*) for body mass (a), body length (b) and parasite induced cataract coverage of the eye lenses (c) before the winter experiment, 27th of October 2020.

**(a)**	ASxAS	ASxLS	LSxAS
LSxLS	**< 0.001**	0.153	0.405
LSxAS	**0.002**	0.946	
ASxLS	**0.014**		

**(b)**	ASxAS	ASxLS	LSxAS
LSxLS	**< 0.001**	0.062	0.245
LSxAS	**0.003**	0.921	
ASxLS	**0.025**		

**(c)**	ASxAS	ASxLS	LSxAS
LSxLS	**< 0.001**	**0.019**	**0.004**
LSxAS	0.173	0.963	
ASxLS	0.058		

Table S1.4. Results of the analysis of variance conducted on salmon body mass (a), body length (b), condition factor (c) and parasite induced cataract coverage of the eye lenses (d) before the summer experiment, 26th of May 2021. For paiwise comparisons of crossing groups in (a), (b), (c) and (d), see table S1.5 below.

**(a) Body mass**	Df	F	P
Crossing group	3	19.97	**< 0.001**
Infection treatment	1	27.47	**< 0.001**
Crossing group × Infection treatment	3	0.01	0.998
**(b) Body length**	Df	F	P
Crossing group	3	16.68	**< 0.001**
Infection treatment	1	31.31	**< 0.001**
Crossing group × Infection treatment	3	0.137	0.938
**(c) Condition factor**	Df	F	P
Crossing group	3	9.13	**< 0.001**
Infection treatment	1	0.97	0.326
Crossing group × Infection treatment	3	0.04	0.991
**(d) Cataract coverage**	Df	F	P
Crossing group	3	10.53	**< 0.001**

Table S1.5 P-values of Tukey-adjusted pairwise Post Hoc comparisons among crossing groups (*Female parent x male parent, LS = Landlocked salmon, AS = Anadromous salmon*) for body mass (a), body length (b), condition factor (c) and parasite induced cataract coverage of the eye lenses (d) before the summer experiment, 26th of May 2021.

**(a)**	ASxAS	ASxLS	LSxAS
LSxLS	**< 0.001**	0.077	**0.028**
LSxAS	**< 0.001**	0.980	
ASxLS	**< 0.001**		
**(b)**	ASxAS	ASxLS	LSxAS
LSxLS	**< 0.001**	0.134	0.188
LSxAS	**< 0.001**	0.998	
ASxLS	**< 0.001**		
**(c)**	ASxAS	ASxLS	LSxAS
LSxLS	**< 0.001**	0.053	**< 0.001**
LSxAS	0.624	0.598	
ASxLS	0.070		
**(d)**	ASxAS	ASxLS	LSxAS
LSxLS	**< 0.001**	0.514	**< 0.001**
LSxAS	0.317	0.156	
ASxLS	**0.001**		

Table S1.6. The number of salmon individuals (n) per predation treatment, crossing group (*Female parent x male parent, LS = Landlocked salmon, AS = Anadromous salmon*) and infection treatment (0 = *uninfected*, 1 = *infected with D. pseudospathaceum*), their mean (± sd) body length (mm), mass (g), condition factor and parasite induced cataract coverage of the eye lenses (%). Table (a) indicates the measurements before the start of the winter experiment, 27th of October 2020 and table (b) gives the measurements after the experiment, 6th April 2021.

(a) Before the winter experiment 27th October 2020

Predation treatment	Strain	Infection treatment	n	Body length	Body mass	Condition factor	Cataract coverage
Control	*LS* x *LS*	0	20	205 ± 17	92.5 ± 22.5	0.99 ± 0.07	-
		1	20	185 ± 21	69.3 ± 22.1	1.02 ± 0.07	42.5 ± 22.7
	LS x *AS*	0	20	192 ± 25	79.5 ± 27.6	1.04 ± 0.07	-
		1	20	179 ± 27	61.6 ± 28.9	0.94 ± 0.17	64.2 ± 23.0
	AS x *LS*	0	21	194 ± 22	81.8 ± 27.4	1.03 ± 0.04	-
		1	20	178 ± 26	63.8 ± 25.6	1.02 ± 0.06	61.2 ± 23.2
	AS x *AS*	0	20	186 ± 18	70.2 ± 18.1	1.02 ± 0.06	-
		1	20	160 ± 27	45.8 ± 22.7	0.98 ± 0.14	65.8 ± 23.7
Predation	*LS* x *LS*	0	20	198 ± 18	84.6 ± 20.9	1.01 ± 0.05	-
		1	20	196 ± 21	80.9 ± 28.3	0.98 ± 0.07	47.5 ± 22.0
	LS x *AS*	0	20	207 ± 19	97.4 ± 27.8	1.01 ± 0.05	-
		1	20	178 ± 23	62.0 ± 22.8	1.02 ± 0.07	61.2 ± 21.6
	AS x *LS*	0	19	194 ± 24	82.2 ± 29.6	1.02 ± 0.04	-
		1	20	179 ± 22	63.3 ± 24.0	1.01 ± 0.09	59.2 ± 28.1
	AS x *AS*	0	20	188 ± 29	75.5 ± 25.2	1.04 ± 0.10	-
		1	20	166 ± 22	47.8 ± 22.6	0.94 ± 0.15	81.0 ± 18.9

(b) After the winter experiment 6th April 2021

Predation treatment	Strain	Infection treatment	n	Body length	Body weight	Condition factor	Cataract coverage
Control	*LS* x *LS*	0	19	217 ± 20	110.0 ± 32.2	0.98 ± 0.05	-
		1	19	193 ± 24	77.9 ± 26.6	0.99 ± 0.05	42.9 ± 23.2
	LS x *AS*	0	20	198 ± 29	85.3 ± 32.6	0.98 ± 0.06	-
		1	20	186 ± 32	68.6 ± 33.5	0.92 ± 0.13	64.2 ± 23.0
	AS x *LS*	0	20	202 ± 27	92.9 ± 37	1.00 ± 0.04	-
		1	20	185 ± 29	71.5 ± 32.6	1.00 ± 0.05	61.2 ± 23.2
	AS x *AS*	0	20	191 ± 19	74.3 ± 21.3	0.98 ± 0.05	-
		1	20	163 ± 29	46.8 ± 24.1	0.96 ± 0.12	65.8 ± 23.7
Predation	*LS* x *LS*	0	13	200 ± 17	79.8 ± 20.4	0.92 ± 0.05	-
		1	12	199 ± 22	78.2 ± 27.1	0.91 ± 0.06	38.3 ± 20.0
	LS x *AS*	0	13	208 ± 21	91.6 ± 25.4	0.93 ± 0.05	-
		1	14	176 ± 27	56.4 ± 24.9	0.94 ± 0.06	55.4 ± 20.9
	AS x *LS*	0	17	189 ± 24	71.7 ± 27.9	0.96 ± 0.04	-
		1	15	180 ± 25	58.3 ± 21.8	0.92 ± 0.06	50.3 ± 26.0
	AS x *AS*	0	19	187 ± 29	68.1 ± 24.2	0.95 ± 0.07	-
		1	7	173 ± 28	51.8 ± 22.7	0.90 ± 0.05	68.6 ± 17.3

Table S1.7. Results of the analysis of variance conducted on change in body mass (a), body length (b) and condition factor (c) of the surviving salmon of the winter experiment, 27th of October 2020 to 6th April 2021. For paiwise comparisons of crossing groups in (a) and (b), see table S1.8 below.

**(a) Change in body mass**	Df	F	P
Crossing group	3	8.95	**< 0.001**
Infection treatment	1	0.00	0.96
Predation treatment	1	365.54	**< 0.001**
Crossing group × Infection treatment	3	2.09	0.102
Crossing group × Predation treatment	3	8.38	**< 0.001**
**(b) Change in body length**	Df	F	P
Crossing group	3	10.90	**< 0.001**
Infection treatment	1	2.94	0.088
Predation treatment	1	328.31	**< 0.001**
Crossing group × Infection treatment	3	0.38	0.768
Crossing group × Predation treatment	3	7.13	**< 0.001**
**(c) Change in condition factor**	Df	F	P
Crossing group	3	1.20	0.310
Infection treatment	1	8.63	**0.003**
Predation treatment	1	104.33	**< 0.001**
Crossing group × Infection treatment	3	2.20	0.089
Crossing group × Predation treatment	3	0.17	0.915

Table S1.8. P-values of Tukey-adjusted pairwise Post Hoc comparisons among crossing groups (*Female parent x male parent, LS = Landlocked salmon, AS = Anadromous salmon*) within the predation-free control tanks for change in body mass (a) and change in body length (b) in the winter experiment, between 27th of October 2020 and 6th April 2021. The crossing groups wthin the predation tanks are ommitted from these comparisons because there were no statistically significant differences among them (see interaction terms in table S1.7).

**(a)**	ASxAS	ASxLS	LSxAS
LSxLS	**< 0.001**	0.097	**0.001**
LSxAS	0.168	0.447	
ASxLS	**0.003**		
**(b)**	ASxAS	ASxLS	LSxAS
LSxLS	**< 0.001**	0.051	**0.006**
LSxAS	**0.004**	0.884	
ASxLS	**0.004**		

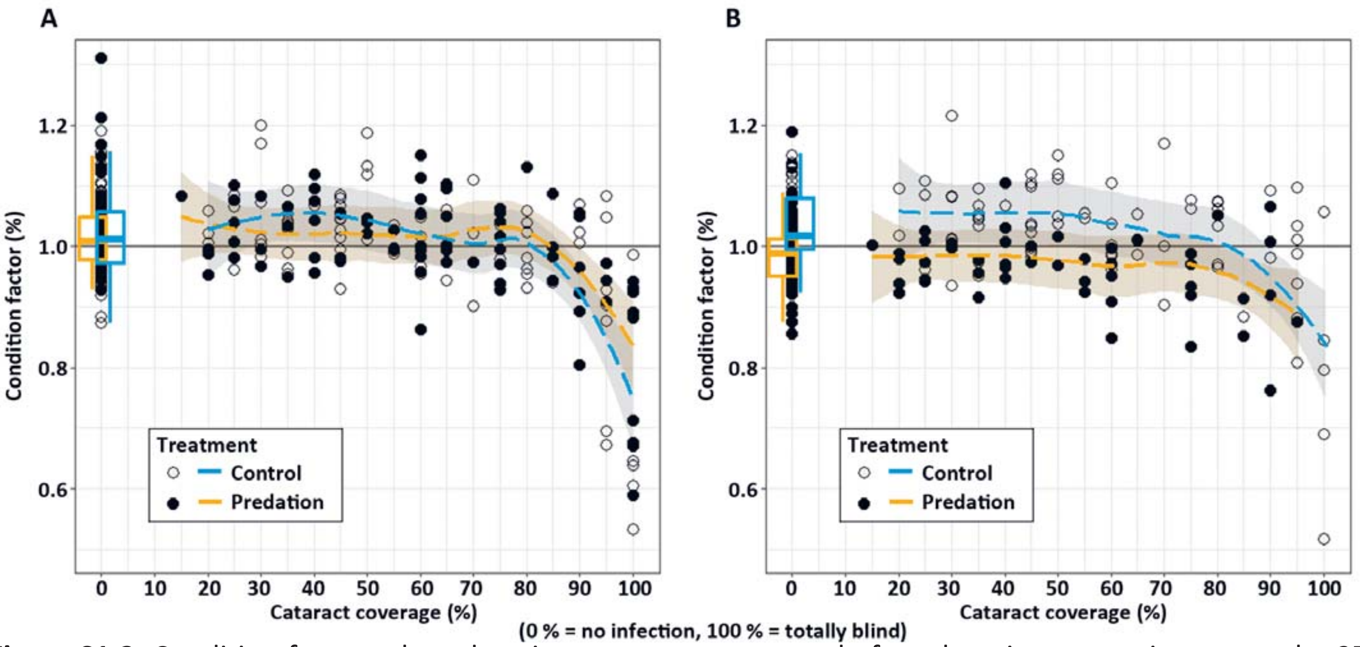

Figure S1.2. Condition factors plotted against cataract coverages before the winter experiment, on the 27th October 2020 (a), and for the surviving fish in the end of the winter experiment, on the 6th April 2021 (b). Empty dots and blue color stand for the control treatment fish and filled circles and yellow color for the predation treatment fish. The boxplots for uninfected fish (cataract coverage 0 %) represent the median condition factors and their interquartile ranges (25th and 75th percentiles), with the whiskers representing 1.5 times the interquartile range. For the infected fish the mean condition factors are represented by dashed loess smoothed splines where the shaded areas give the 0.95 confidence intervals.

Appendix S2 The Model selection process leading to the mortality model in Table 2a:
All salmon of the predation treatment (accounting for infection, 0/1)

STEP 1. The candidate models:

```
CauseofDeathPredation = The binomial response variable (survived/depredated)
Infection = Infection treatment (0 = Uninfected, 1 = Infected with D. pseudospathaceum)
Strain = Crossing group (LSxLS, LSxAS, ASxLS, ASxAS)
Strain2 = Crossing group (Landlocked, Hybrid, Anadromous)
Trial = Experiment (Winter, Summer)
Tanktrial = Tank, used as random factor (four predation tanks in both experiments)
+ = main effect
* = main effect and interaction
-----------------------------------------------------------------------------------------
A1.1 <- glmmTMB(CauseofDeathPredation ~ Infection * Strain * Trial + (1|Tanktrial),
               data = Predated, family = "betabinomial"(link = "logit"))

A1.2 <- glmmTMB(CauseofDeathPredation ~ Infection * Strain + Trial + (1|Tanktrial),
              data = Predated, family = "betabinomial"(link = "logit"))

A1.3 <- glmmTMB(CauseofDeathPredation ~ Infection + Strain * Trial + (1|Tanktrial),
                data = Predated, family = "betabinomial"(link = "logit"))

A1.4 <- glmmTMB(CauseofDeathPredation ~ Infection + Strain + Trial + (1|Tanktrial),
                data = Predated, family = "betabinomial"(link = "logit"))

A1.5 <- glmmTMB(CauseofDeathPredation ~ Infection * Strain + (1|Tanktrial),
                data = Predated, family = "betabinomial"(link = "logit"))

A1.6 <- glmmTMB(CauseofDeathPredation ~ Infection * Trial + (1|Tanktrial),
                data = Predated, family = "betabinomial"(link = "logit"))

A1.7 <- glmmTMB(CauseofDeathPredation ~ Infection + Strain + (1|Tanktrial),
                data = Predated, family = "betabinomial"(link = "logit"))

A1.8 <- glmmTMB(CauseofDeathPredation ~ Infection + Trial + (1|Tanktrial),
                data = Predated, family = "betabinomial"(link = "logit"))

A1.9 <- glmmTMB(CauseofDeathPredation ~ Infection + (1|Tanktrial),
                data = Predated, family = "betabinomial"(link = "logit"))

A1.10 <- glmmTMB(CauseofDeathPredation ~ Strain + (1|Tanktrial),
                data = Predated, family = "betabinomial"(link = "logit"))

A1.11 <- glmmTMB(CauseofDeathPredation ~ Trial + (1|Tanktrial),
                data = Predated, family = "betabinomial"(link = "logit"))

A2.1 <- glmmTMB(CauseofDeathPredation ~ Infection * Strain2 * Trial + (1|Tanktrial),
                 data = Predated, family = "betabinomial"(link = "logit"))

A2.2 <- glmmTMB(CauseofDeathPredation ~ Infection * Strain2 + Trial + (1|Tanktrial),
                 data = Predated, family = "betabinomial"(link = "logit"))

A2.3 <- glmmTMB(CauseofDeathPredation ~ Infection + Strain2 * Trial + (1|Tanktrial),
                 data = Predated, family = "betabinomial"(link = "logit"))

A2.4 <- glmmTMB(CauseofDeathPredation ~ Infection + Strain2 + Trial + (1|Tanktrial),
                 data = Predated, family = "betabinomial"(link = "logit"))

A2.5 <- glmmTMB(CauseofDeathPredation ~ Infection * Strain2+ (1|Tanktrial),
                 data = Predated, family = "betabinomial"(link = "logit"))

A2.6 <- glmmTMB(CauseofDeathPredation ~ Infection + Strain2 + (1|Tanktrial),
                 data = Predated, family = "betabinomial"(link = "logit"))

A2.7 <- glmmTMB(CauseofDeathPredation ~ Strain2 + (1|Tanktrial),
                  data = Predated, family = "betabinomial"(link = "logit"))

Models A1.2 and A2.1 failed to converge (due to a non-positive-definite Hessian matrix).
```

STEP 2. Evaluating all candidate models against each other:

Table S2.1 X^2 and corresponding $p > \chi^2$- values (with $p < 0.05$ highlighted in bold) of pairwise comparisons of all candidate models (see previous page for the model codes). The first row and column show the AIC-values of the candidate model in bold under the model's code (N.A. means that there were convergence problems and no AIC-value could be assigned). The three models with the lowest AIC-values (A1.1, A2.5 and A2.2) and their pairwise tests are highlighted in grey. Note that these three models did not differ significantly from each other ($p < 0.05$) and, thus, model A2.2 was chosen as the final model, since we wanted to show that the summer and winter experiments did not differ.

	A1.1<br>**350.1**	A1.2<br>N.A.	A1.3<br>**365.0**	A1.4<br>**361.1**	A1.5<br>**388.9**	A1.6<br>**362.3**	A1.7<br>**359.1**	A1.8<br>**360.4**	A1.9<br>**358.4**	A1.10<br>**370.8**	A1.11<br>**371.8**	A2.1<br>N.A.	A2.2<br>**350.1**	A2.3<br>**363.2**	A2.4<br>**359.7**	A2.5<br>**348.2**	A2.6<br>**357.7**
A2.7<br>**369.4**	**42.74**<br>**<0.001**		**16.38**<br>**0.012**	**14.31**<br>**0.003**	**28.17**<br>**<0.001**	**9.07**<br>**0.003**	**14.27**<br>**<0.001**	0.00<br>1.000	0.00<br>1.000	0.58<br>0.447	**4.38**<br>**0.036**		**27.26**<br>**<0.001**	**14.20**<br>**0.007**	**13.69**<br>**0.001**	**27.22**<br>**<0.001**	**27.22**<br>**<0.001**
A2.6<br>**357.7**	**29.09**<br>**0.004**		2.73<br>0.742	0.66<br>0.719	**14.52**<br>**0.006**	**4.58**<br>**<0.001**	0.62<br>0.431	**4.63**<br>**0.031**	4.67<br>0.097	**13.07**<br>**<0.001**	**18.03**<br>**<0.001**		**13.61**<br>**0.003**	0.55<br>0.907	0.04<br>0.846	**13.57**<br>**0.001**	
A2.5<br>**348.2**	15.52<br>0.114		0.00<br>1.000	**12.91**<br>**<0.001**	0.95<br>0.622	**18.15**<br>**<0.001**	**12.95**<br>**<0.001**	**18.20**<br>**<0.001**	**18.24**<br>**0.001**	**26.64**<br>**<0.001**	**31.60**<br>**<0.001**		0.04<br>0.843	0.00<br>1.000	**13.53**<br>**<0.001**		
A2.4<br>**359.7**	**29.05**<br>**0.002**		2.69<br>0.611	0.621<br>0.431	**14.48**<br>**0.002**	**4.62**<br>**0.032**	0.00<br>1.000	4.67<br>0.097	4.71<br>0.194	**13.11**<br>**<0.001**	**18.07**<br>**<0.001**		**13.57**<br>**0.001**	0.51<br>0.773			
A2.3<br>**363.2**	**28.54**<br>**<0.001**		2.17<br>0.337	0.00<br>1.000	**13.96**<br>**<0.001**	5.13<br>0.162	0.00<br>1.000	5.19<br>0.269	5.22<br>0.389	**13.62**<br>**0.003**	**18.59**<br>**0.002**		0.00<br>1.000				
A2.2<br>**350.1**	15.49<br>0.080		0.00<br>1.000	**12.95**<br>**<0.001**	0.911<br>0.340	**18.19**<br>**<0.001**	**12.98**<br>**0.002**	**18.24**<br>**0.001**	**18.28**<br>**0.002**	**26.68**<br>**<0.001**	**31.64**<br>**<0.001**						
A2.1<br>N.A.																	
A1.11<br>**371.8**	**47.12**<br>**<0.001**		**20.76**<br>**0.004**	**18.69**<br>**0.001**	**32.55**<br>**<0.001**	**13.45**<br>**0.001**	**18.65**<br>**<0.001**	**13.40**<br>**<0.001**	0.00<br>1.000	4.96<br>0.084							
A1.10<br>**370.8**	**42.16**<br>**<0.001**		**15.80**<br>**0.007**	**13.73**<br>**0.001**	**27.59**<br>**<0.001**	0.00<br>1.000	**13.69**<br>**<0.001**	0.00<br>1.000	0.00<br>1.000								
A1.9<br>**358.4**	**33.76**<br>**0.002**		7.40<br>0.389	5.33<br>0.255	**19.19**<br>**0.004**	0.09<br>0.956	5.29<br>0.152	0.04<br>0.847									
A1.8<br>**360.4**	**33.72**<br>**0.001**		7.36<br>0.289	5.29<br>0.152	**19.15**<br>**0.002**	0.05<br>0.818	5.25<br>0.072										
A1.7<br>**359.1**	**28.47**<br>**0.003**		2.11<br>0.716	0.03<br>0.846	**13.90**<br>**0.003**	**5.20**<br>**0.023**											
A1.6<br>**362.3**	**33.67**<br>**<0.001**		7.31<br>0.199	5.24<br>0.073	**19.10**<br>**<0.001**												
A1.5<br>**388.9**	14.57<br>0.068		0.00<br>1.000	**13.86**<br>**0.001**													
A1.4<br>**361.1**	**28.43**<br>**0.002**		2.07<br>0.558														
A1.3<br>**365.0**	**26.36**<br>**<0.001**																

STEP 3. Choosing a variance structure for the final model:

```
CauseofDeathPredation = The binomial response variable (survived/depredated)
Infection = Infection treatment (0 = Uninfected, 1 = Infected with D. pseudospathaceum)
Strain = Crossing group (LSxLS, LSxAS, ASxLS, ASxAS)
Strain2 = Crossing group (Landlocked, Hybrid, Anadromous)
Trial = Experiment (Winter, Summer)
Tanktrial = Tank, used as random factor (four predation tanks in both experiments)
+ = main effect
* = main effect and interaction
-------------------------------------------------------------------------------------------
# Model with no random variance at all:
A2.2.1 <- glmmTMB(CauseofDeathPredation ~ Infection * Strain2 + Trial,
                  data = Predated, family = "betabinomial"(link = "logit"))

# Individual variances per tank:
A2.2.2 <- glmmTMB(CauseofDeathPredation ~ Infection * Strain2 + Trial + (1|Tanktrial),
                  data = Predated, family = "betabinomial"(link = "logit"))

# Individual variances per tank, nested within experiment.
A2.2.3 <- glmmTMB(CauseofDeathPredation ~ Infection * Strain2 + (Trial|Tanktrial),
                  data = Predated, family = "betabinomial"(link = "logit"))

# Model A2.2.3 failed to converge and had to be excluded from the final model comparison.
```

Testing for AIC-values:

	Df	AIC	BIC	logLik	deviance	Chisq	Chi Df	Pr(>Chisq)	
A2.2.1	8	371.38	401.50	-177.69	355.38				
**A2.2.2**	**9**	**350.11**	**384.00**	**-166.06**	**332.11**	**23.2648**	**1**	**1.412e-06**	*******

→ Model A2.2.2 (highlighted in bold) with individual variances per tank is chosen as the final model due to lowest AIC-value.

STEP 4. Residual diagnostics of the final model (based on simulated residuals):

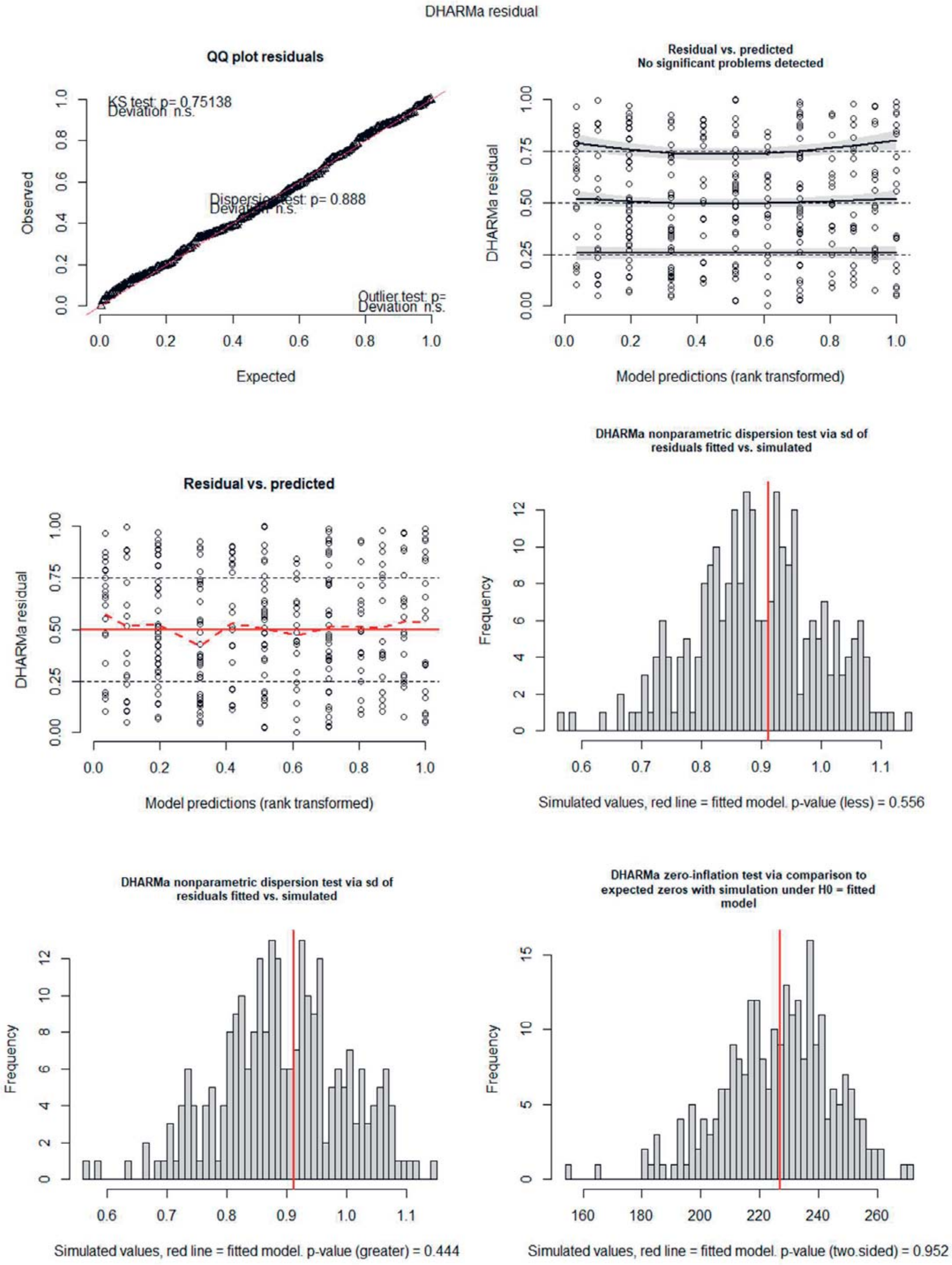

Appendix S3 The Model selection process leading to the mortality model in Table 2b:
Only uninfected salmon of the predation treatment (infection 0)

STEP 1. The candidate models:

```
Model B involves only unininfected salmon (hence the data "UninfectedPredated")

CauseofDeathPredation = The binomial response variable (survived/depredated)
Strain2 = Crossing group (Landlocked, Hybrid, Anadromous)
   To be consistent with the previous model (Appendix S2), we will use only Strain2
Condition_start = Condition factor in the start of the experiment.
   Trying to use length or weight as covariates led to convergence problems, but the condition
   factors could be used…
Trial = Experiment (Winter, Summer)
Tanktrial = Tank, used as random factor (four predation tanks in both experiments)
+ = main effect
* = main effect and interaction
-----------------------------------------------------------------------------------------
B1 <- glmmTMB(CauseofDeathPredation ~ Condition_start * Strain2 * Trial + (1|Tanktrial),
                data = UninfectedPredated, family = "betabinomial"(link = "logit"))

B2 <- glmmTMB(CauseofDeathPredation ~ Condition_start + Strain2 * Trial + (1|Tanktrial),
                data = UninfectedPredated, family = "betabinomial"(link = "logit"))

B3 <- glmmTMB(CauseofDeathPredation ~ Condition_start * Strain2 + Trial + (1|Tanktrial),
                data = UninfectedPredated, family = "betabinomial"(link = "logit"))

B4 <- glmmTMB(CauseofDeathPredation ~ Condition_start * Trial + (1|Tanktrial),
                data = UninfectedPredated, family = "betabinomial"(link = "logit"))

B5 <- glmmTMB(CauseofDeathPredation ~ Condition_start + Strain2 + (1|Tanktrial),
                data = UninfectedPredated, family = "betabinomial"(link = "logit"))

B6 <- glmmTMB(CauseofDeathPredation ~ Condition_start + Trial + (1|Tanktrial),
                 data = UninfectedPredated, family = "betabinomial"(link = "logit"))

B7 <- glmmTMB(CauseofDeathPredation ~ Strain2 * Trial + (1|Tanktrial),
                 data = UninfectedPredated, family = "betabinomial"(link = "logit"))

B8 <- glmmTMB(CauseofDeathPredation ~ Condition_start + (1|Tanktrial),
                 data = UninfectedPredated, family = "betabinomial"(link = "logit"))

B9 <- glmmTMB(CauseofDeathPredation ~ Strain2 + Trial + (1|Tanktrial),
              data = UninfectedPredated, family = "betabinomial"(link = "logit"))

B10 <- glmmTMB(CauseofDeathPredation ~ Strain2 + (1|Tanktrial),
              data = UninfectedPredated, family = "betabinomial"(link = "logit"))

B11 <- glmmTMB(CauseofDeathPredation ~ Trial + (1|Tanktrial),
               data = UninfectedPredated, family = "betabinomial"(link = "logit"))

Models B9, B10 and B11 failed to converge and had to be excluded from the AIC-comparisons.
```

STEP 2. Comparing the AIC-values of the candidate models:

Testing for AIC-values:

```
   Df    AIC    BIC  logLik deviance  Chisq Chi Df Pr(>Chisq)    
B8  4 157.15 169.42 -74.573   149.15                             
B6  5 158.95 174.29 -74.473   148.95 0.2011      1    0.65387    
B4  6 157.39 175.81 -72.696   145.39 3.5537      1    0.05941 .  
B5  6 152.15 170.56 -70.073   140.15 5.2450      0    < 2e-16 ***
B7  8 151.17 175.72 -67.587   135.17 4.9732      2    0.08319 .  
B2  9 152.53 180.15 -67.263   134.53 0.6484      1    0.42068    
B3  9 157.60 185.22 -69.799   139.60 0.0000      0    1.00000    
B1 14 158.51 201.48 -65.256   130.51 9.0857      5    0.10569    
```

Model B7 has the lowest AIC-value, albeit it was not significantly different from model B5.

STEP 3. Choosing a variance structure for the final model:

```
# Model with no random variance at all:
B7.1 <- glmmTMB(CauseofDeathPredation ~ Strain2 * Trial,
                  data = UninfectedPredated, family = "betabinomial"(link = "logit"))

# Individual variances per tank:
B7.2 <- glmmTMB(CauseofDeathPredation ~ Strain2 * Trial + (1|Tanktrial),
                  data = UninfectedPredated, family = "betabinomial"(link = "logit"))

# Individual variances per tank, nested within experiment:
B7.3 <- glmmTMB(CauseofDeathPredation ~ Strain2 + (Trial|Tanktrial),
                  data = UninfectedPredated, family = "betabinomial"(link = "logit"))
```

Testing for AIC-values:

```
     Df    AIC    BIC  logLik deviance  Chisq Chi Df Pr(>Chisq)    
B7.1  7 161.45 182.94 -73.727   147.45                             
B7.3  7 148.50 169.99 -67.252   134.50 12.951      0     <2e-16 ***
B7.2  8 151.17 175.72 -67.587   135.17  0.000      1          1    
```

→ Model B7.3 (highlighted in bold), with individual variances per tank, nested within experiment, has the lowest AIC-value.

STEP 4. Residual diagnostics of the final models (based on simulated residuals):

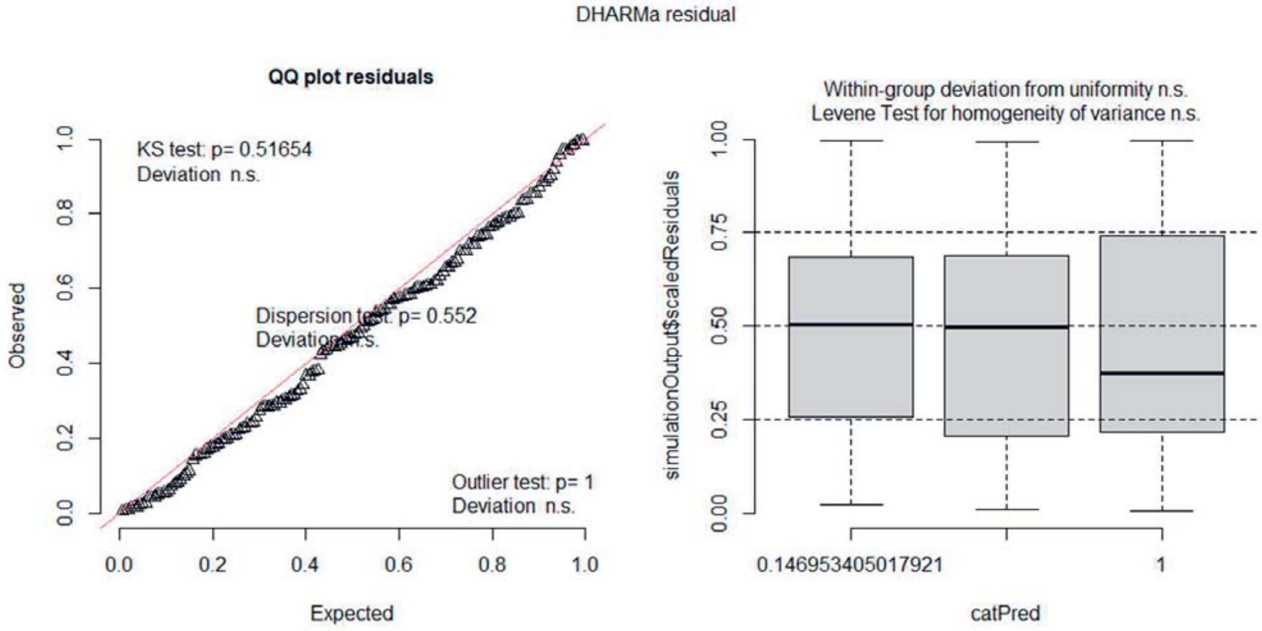

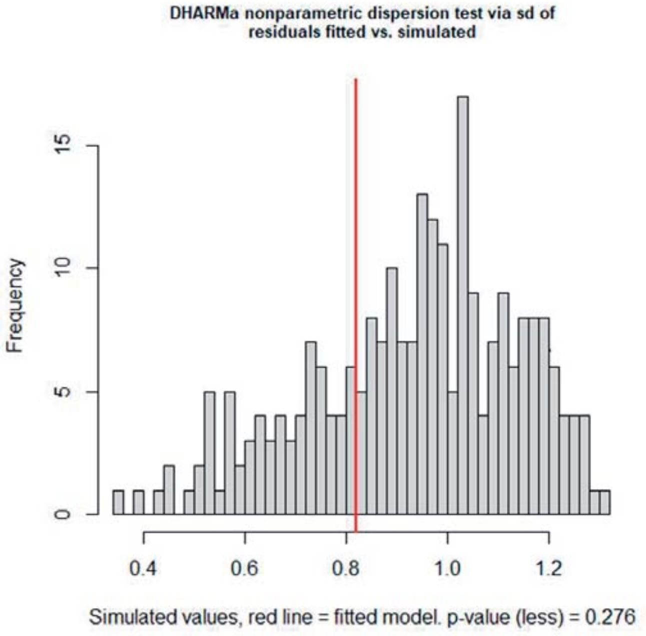

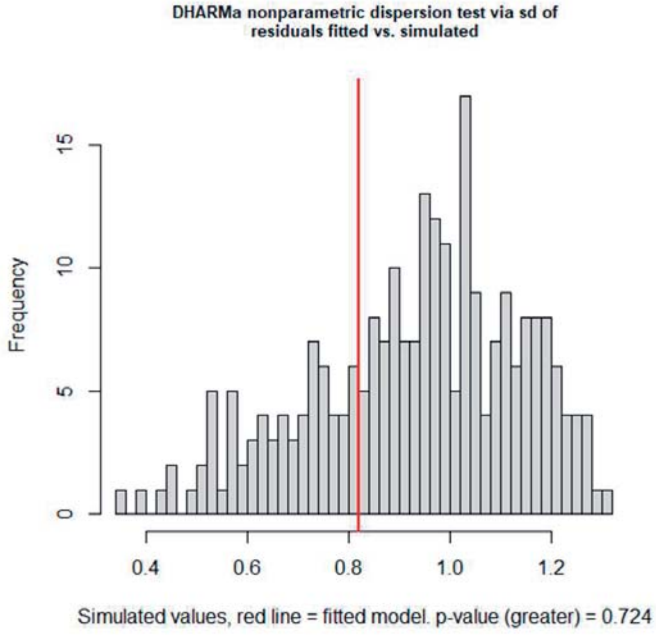

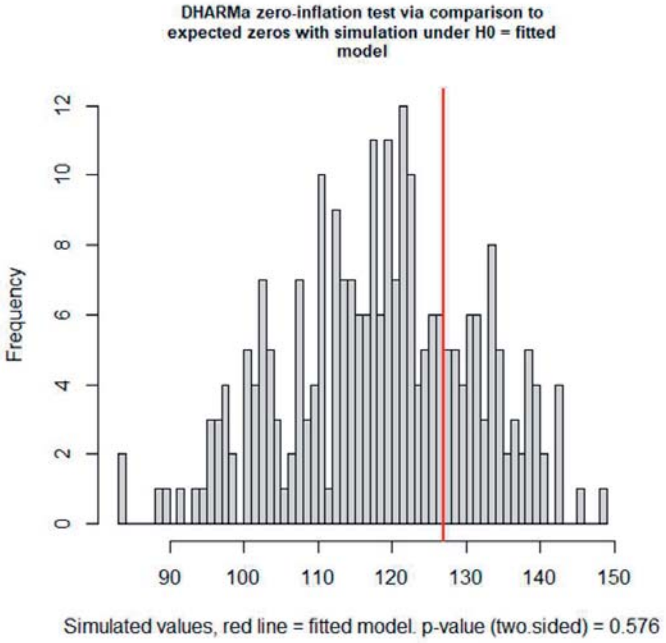

Appendix S4 The Model selection process leading to the mortality model in Table 2c: *Only infected salmon of the predation treatment (infection status 1)*

STEP 1. The candidate models:

```
Model C involves only infected salmon (hence the data "InfectedPredated")

CauseofDeathPredation = The binomial response variable (survived/depredated)
DegreeofBlindness = Coverage of parasite-induced cataracts in the eye lenses
Strain2 = Crossing group (Landlocked, Hybrid, Anadromous)
   To be consistent with the previous models (Appendix S2, S3), we will use only Strain2
Trial = Experiment (Winter, Summer)
Tanktrial = Tank, used as random factor (four predation tanks in both experiments)
+ = main effect
* = main effect and interaction
-----------------------------------------------------------------------------------------
C1 <- glmmTMB(CauseofDeathPredation ~ DegreeofBlindness * Strain2 * Trial + (1|Tanktrial),
                data = InfectedPredated, family = "betabinomial"(link = "logit"))

C2 <- glmmTMB(CauseofDeathPredation ~ DegreeofBlindness * Strain2 + Trial + (1|Tanktrial),
              data = InfectedPredated, family = "betabinomial"(link = "logit"))

C3 <- glmmTMB(CauseofDeathPredation ~ DegreeofBlindness + Strain2 * Trial + (1|Tanktrial),
                data = InfectedPredated, family = "betabinomial"(link = "logit"))

C4 <- glmmTMB(CauseofDeathPredation ~ DegreeofBlindness + Strain2 + Trial + (1|Tanktrial),
                data = InfectedPredated, family = "betabinomial"(link = "logit"))

C5 <- glmmTMB(CauseofDeathPredation ~ DegreeofBlindness * Strain2 + (1|Tanktrial),
                data = InfectedPredated, family = "betabinomial"(link = "logit")) #

C6 <- glmmTMB(CauseofDeathPredation ~ DegreeofBlindness * Trial + (1|Tanktrial),
                data = InfectedPredated, family = "betabinomial"(link = "logit"))

C7 <- glmmTMB(CauseofDeathPredation ~ DegreeofBlindness + Strain2 + (1|Tanktrial),
                data = InfectedPredated, family = "betabinomial"(link = "logit"))

C8 <- glmmTMB(CauseofDeathPredation ~ DegreeofBlindness + Trial + (1|Tanktrial),
                data = InfectedPredated, family = "betabinomial"(link = "logit"))

C9 <- glmmTMB(CauseofDeathPredation ~ DegreeofBlindness + (1|Tanktrial),
                data = InfectedPredated, family = "betabinomial"(link = "logit"))

C10 <- glmmTMB(CauseofDeathPredation ~ Strain2 + (1|Tanktrial),
                 data = InfectedPredated, family = "betabinomial"(link = "logit"))

C11 <- glmmTMB(CauseofDeathPredation ~ Trial + (1|Tanktrial),
                 data = InfectedPredated, family = "betabinomial"(link = "logit"))

Model C5 failed to converge (non-positive-definite Hessian matrix) and had to be excluded from
the AIC comparisons.
```

STEP 2. Comparing the AIC-values of the candidate models:

```
Testing for AIC-values:

    Df    AIC    BIC   logLik deviance   Chisq Chi Df Pr(>Chisq)
C9   4 178.29 190.59  -85.144   170.29
C11  4 216.29 228.59 -104.146   208.29  0.0000      0     1.0000
C8   5 180.28 195.66  -85.142   170.28 38.0064      1  7.051e-10 ***
C10  5 208.73 224.11  -99.366   198.73  0.0000      0     1.0000
C6   6 182.14 200.59  -85.071   170.14 28.5899      1  8.945e-08 ***
C7   6 179.41 197.86  -83.702   167.41  2.7371      0  < 2.2e-16 ***
C4   7 181.40 202.92  -83.698   167.40  0.0089      1     0.9247
C2   9 184.94 212.62  -83.470   166.94  0.4560      2     0.7961
C1  14 189.95 233.01  -80.977   161.95  4.9857      5     0.4176

The lowest AIC-value was found in model C9, but it was not significantly different from C7:

   Df    AIC    BIC  logLik deviance Chisq Chi Df Pr(>Chisq)
C9  4 178.29 190.59 -85.144   170.29
C7  6 179.41 197.86 -83.702   167.41 2.883      2     0.2366

Since we are interested in the possible effect of crossing group, we will choose model C7.
```

STEP 3. Choosing a variance structure for the final model:

```
# Model with no random variance at all:
C7.1 <- glmmTMB(CauseofDeathPredation ~ DegreeofBlindness + Strain2,
                   data = InfectedPredated, family = "betabinomial"(link = "logit"))

# Individual variances per tank:
C7.2 <- glmmTMB(CauseofDeathPredation ~ DegreeofBlindness + Strain2 + (1|Tanktrial),
                  data = InfectedPredated, family = "betabinomial"(link = "logit"))

# Individual variances per tank, nested within experiment:
C7.3 <- glmmTMB(CauseofDeathPredation ~ DegreeofBlindness + Strain2 + (Tank|Tanktrial),
                   data = InfectedPredated, family = "betabinomial"(link = "logit"))
```

→ Models C7.2 and C7.3 failed to converge and thus model C7.2 was the only possibility.
.

STEP 4. Residual diagnostics of the final model (based on simulated residuals):

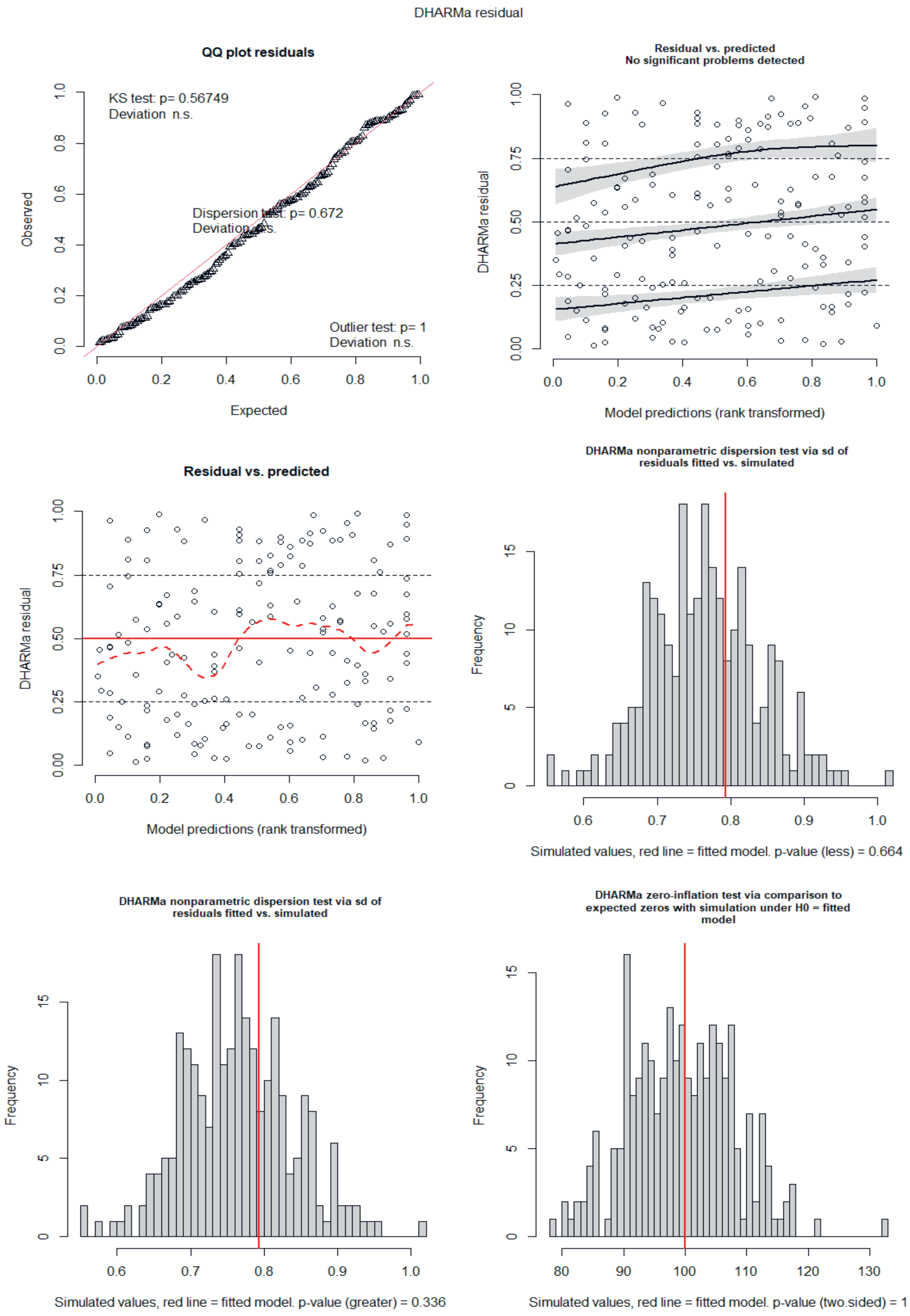

Appendix S3 Movement activity of the fish during the experiments:

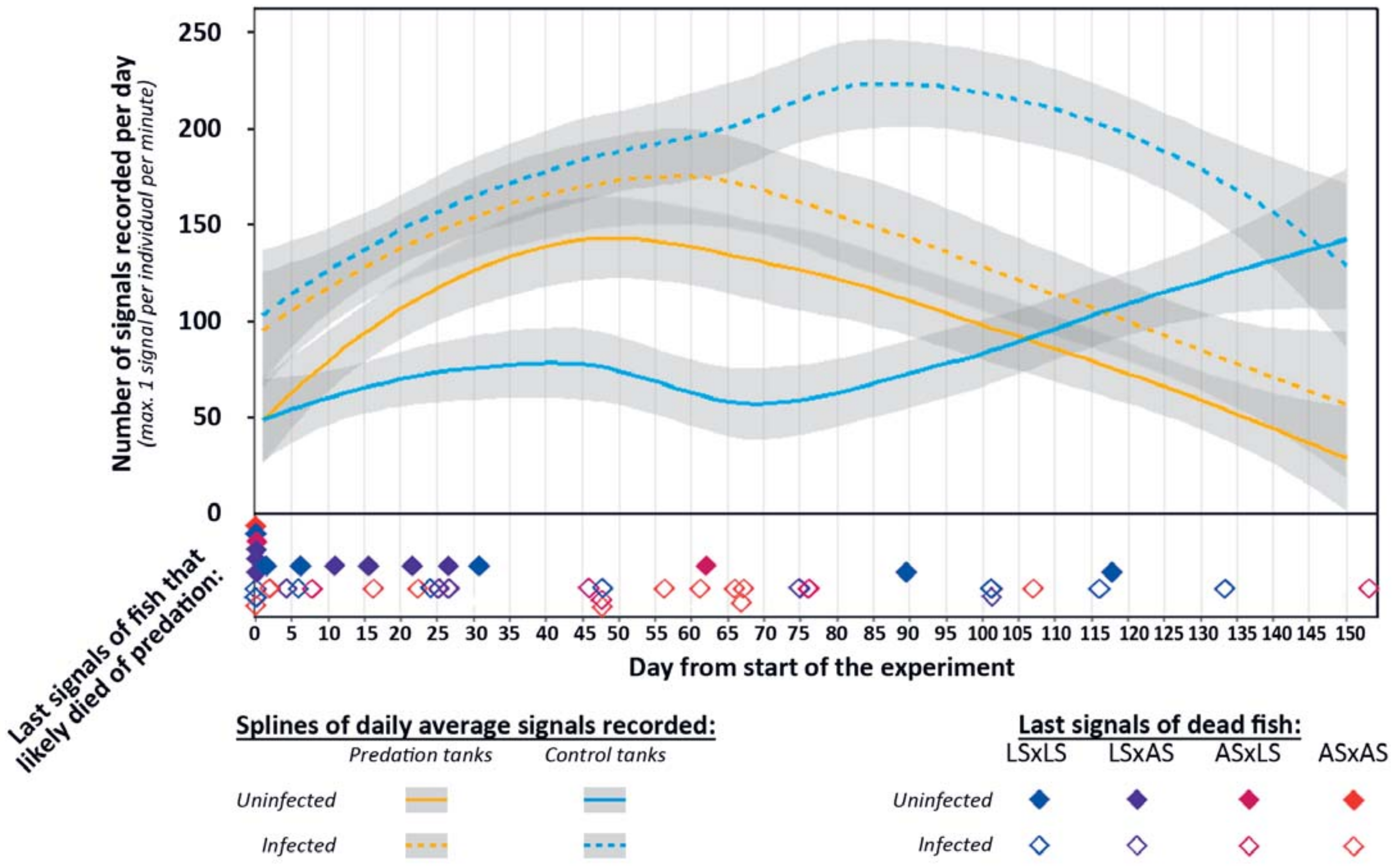

Figure S3.1 Relative movement activity of the fish during the winter experiment and suspected mortality events. The *y*-axis gives the number of signals recorded by the PIT-antennae per day and the *x*-axis gives the time in days from the start of the experiment (3rd of November 2020 to 6th of April 2021). The loess smoothed splines represent relative movement activity of the fish, given as the average number of signals recorded in the respective treatments per day (blue for the four control tanks, yellow for the four predation tanks), divided into signals recorded from healthy fish (continuous lines) and signals recorded from fish infected with *D. pseudospathaceum* (dashed lines). These signals were restricted to a maximum of one signal per individual per minute to avoid overplotting. The last signals recorded for fish that died due to predation are shown as diamonds. Here, filled diamonds imply uninfected fish and empty diamonds infected fish, while different colors represent different crossing groups: *LS = Landlocked salmon, AS = Anadromous salmon, Female parent x male parent.*

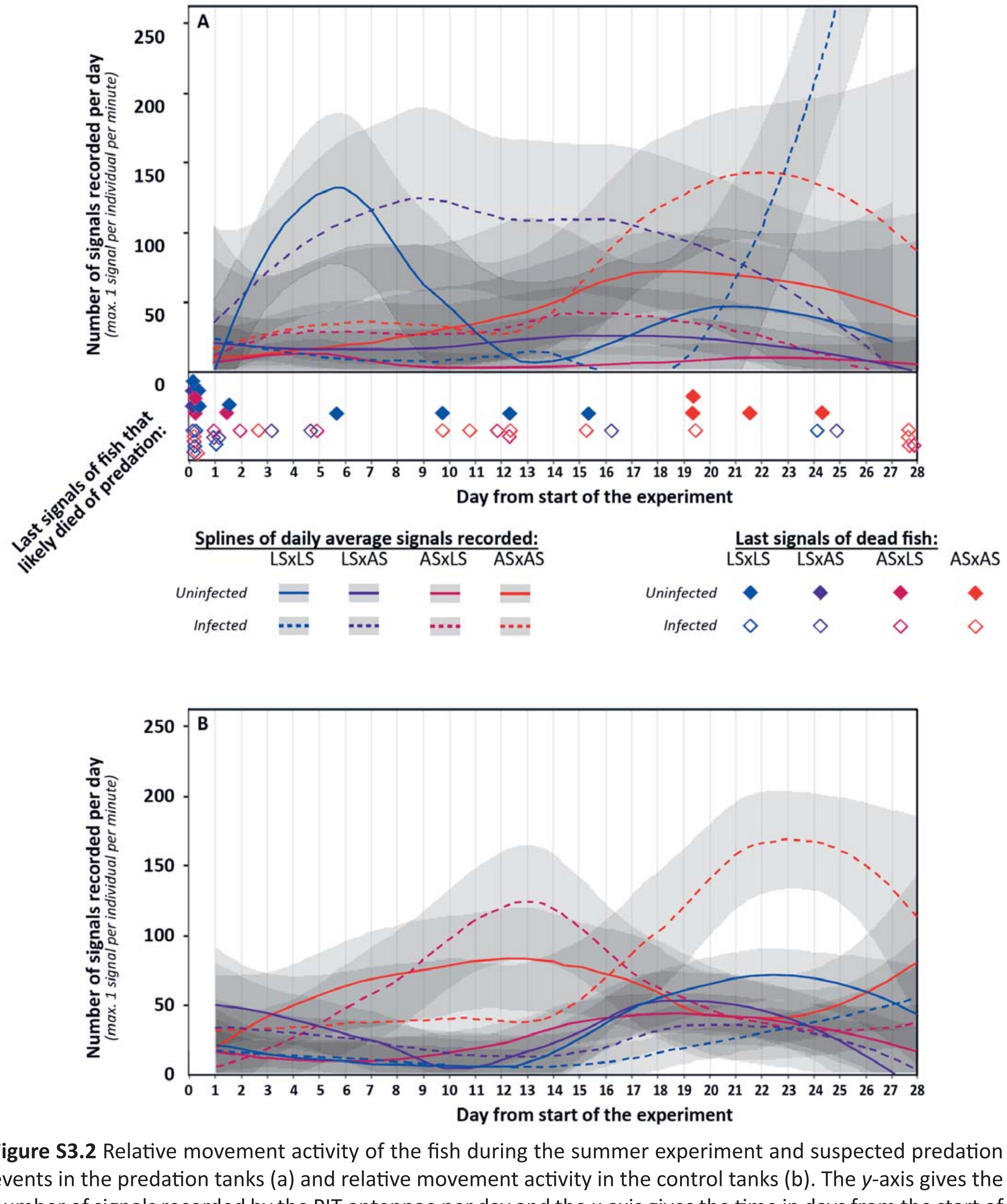

Figure S3.2 Relative movement activity of the fish during the summer experiment and suspected predation events in the predation tanks (a) and relative movement activity in the control tanks (b). The *y*-axis gives the number of signals recorded by the PIT-antennae per day and the *x*-axis gives the time in days from the start of the experiment (30th of May to 26th of June 2021). The loess smoothed splines represent relative movement activity of the fish, given as the average number of signals recorded in the respective treatments per day (with colors representing different crossing groups: *LS = Landlocked salmon, AS = Anadromous salmon, Female parent x male parent.*), divided into signals recorded from healthy fish (continuous lines) and signals recorded from fish infected with *D. pseudospathaceum* (dashed lines). These signals were restricted to a maximum of one signal per individual per minute to avoid overplotting. The last signals recorded for fish that died due to predation in (b) are shown as diamonds. Here, filled diamonds imply healthy fish and empty diamonds infected fish.

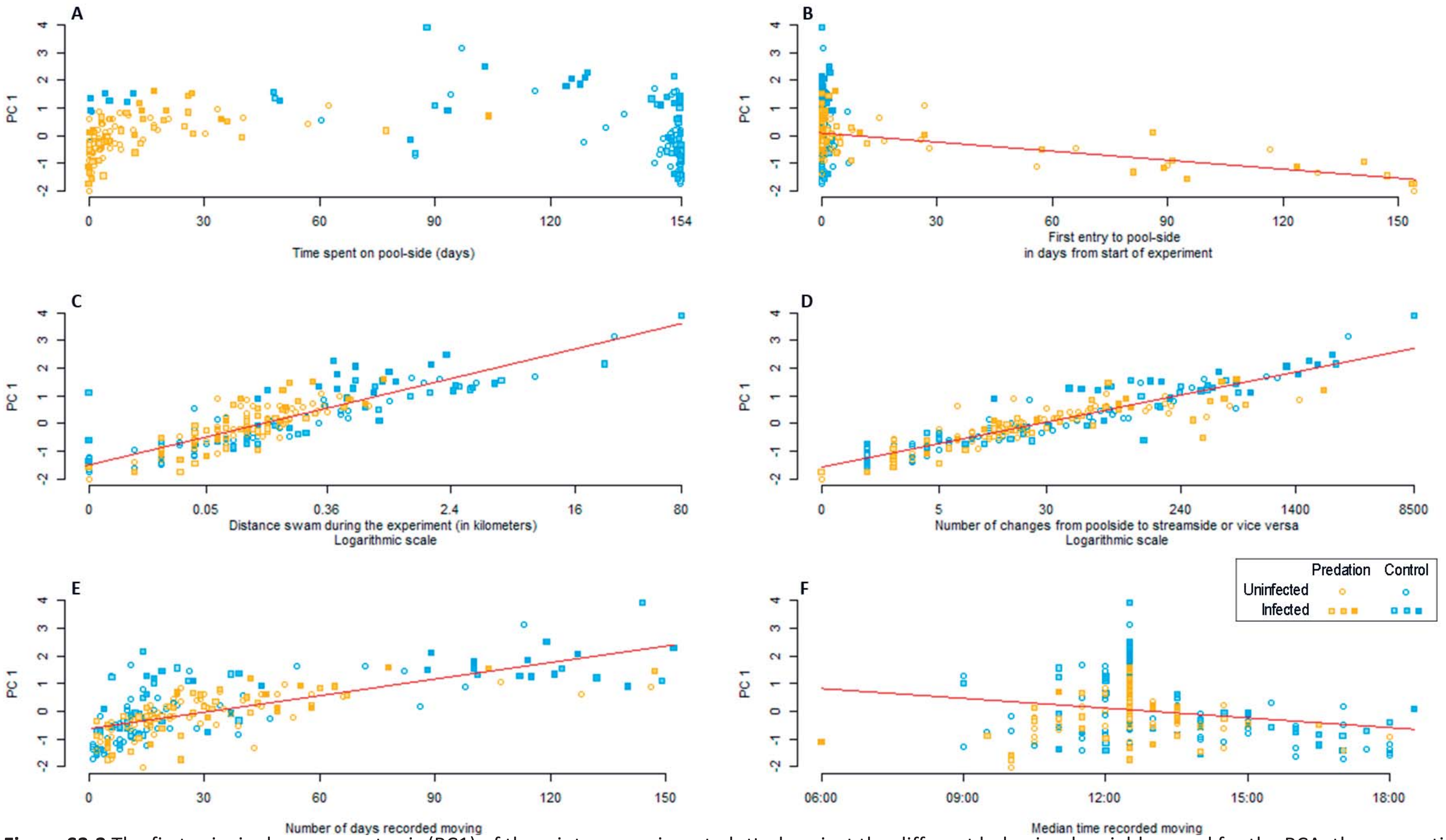

Figure S3.3 The first principal component axis (PC1) of the winter experiment plotted against the different behavioral variables used for the PCA: the proportion of time spent in the pool section of the tanks in days (a), the first entry to the pool-side section in days (b), the total distance swam during the experiment on a logarithmic scale in kilometers (c), the number of changes between the stream- and pool-sections on a logarithmic scale (d), the number of days on which an individual was recorded (e), and the median clock hour of the day the individuals were moving (f). Individuals in predation tanks are represented by yellow symbols and control tank individuals by blue symbols, where empty circles represent uninfected individuals and squares represent infected individuals. Statistically significant correlations as red lines (p <0.001).

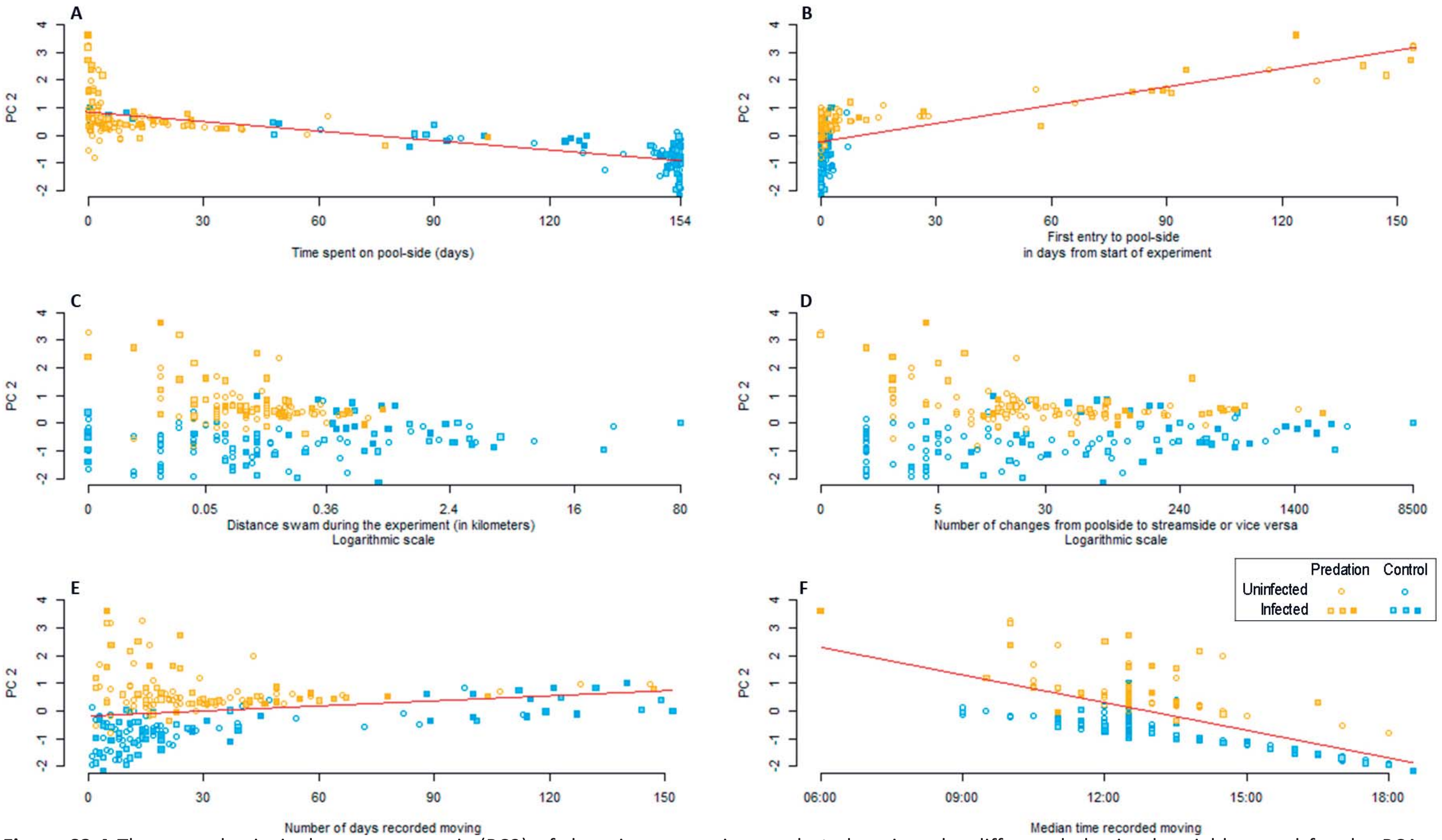

Figure S3.4 The second principal component axis (PC2) of the winter experiment plotted against the different behavioral variables used for the PCA: the proportion of time spent in the pool section of the tanks in days (a), the first entry to the pool-side section in days (b), the total distance swam during the experiment on a logarithmic scale in kilometers (c), the number of changes between the stream- and pool-sections on a logarithmic scale (d), the number of days on which an individual was recorded (e), and the median clock hour of the day the individuals were moving (f). Individuals in predation tanks are represented by yellow symbols and control tank individuals by blue symbols, where empty points represent uninfected individuals and squares represent infected individuals. Statistically significant correlations as red lines (p <0.001).

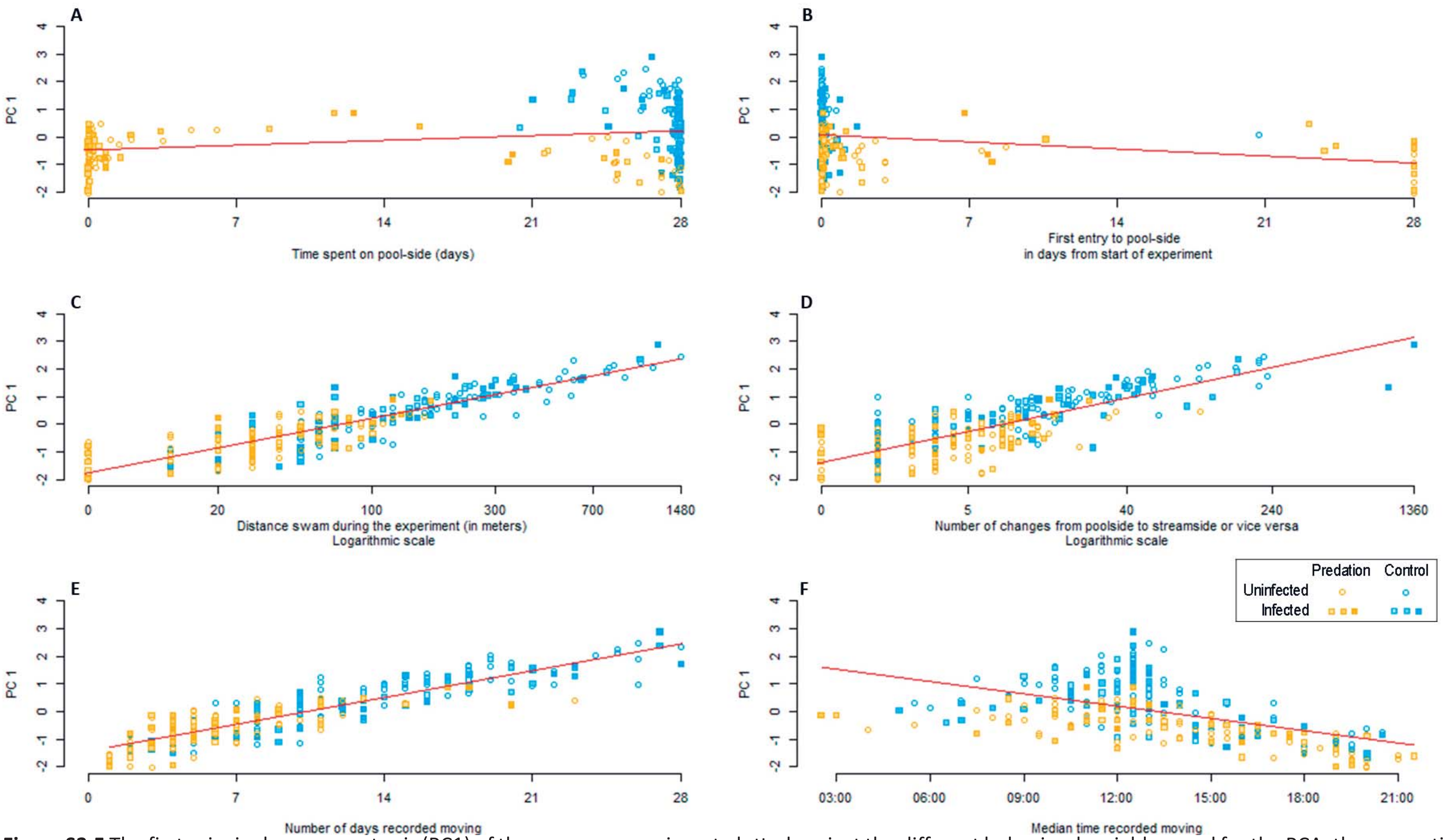

Figure S3.5 The first principal component axis (PC1) of the summer experiment plotted against the different behavioral variables used for the PCA: the proportion of time spent in the pool section of the tanks in days (a), the first entry to the pool-side section in days (b), the total distance swam during the experiment on a logarithmic scale in meters (c), the number of changes between the stream- and pool-sections on a logarithmic scale (d), the number of days on which an individual was recorded (e), and the median clock hour of the day the individuals were moving (f). Individuals in predation tanks are represented by yellow symbols and control tank individuals by blue symbols, where empty points represent uninfected individuals and squares represent infected individuals. Statistically significant correlations as red lines (p <0.001).

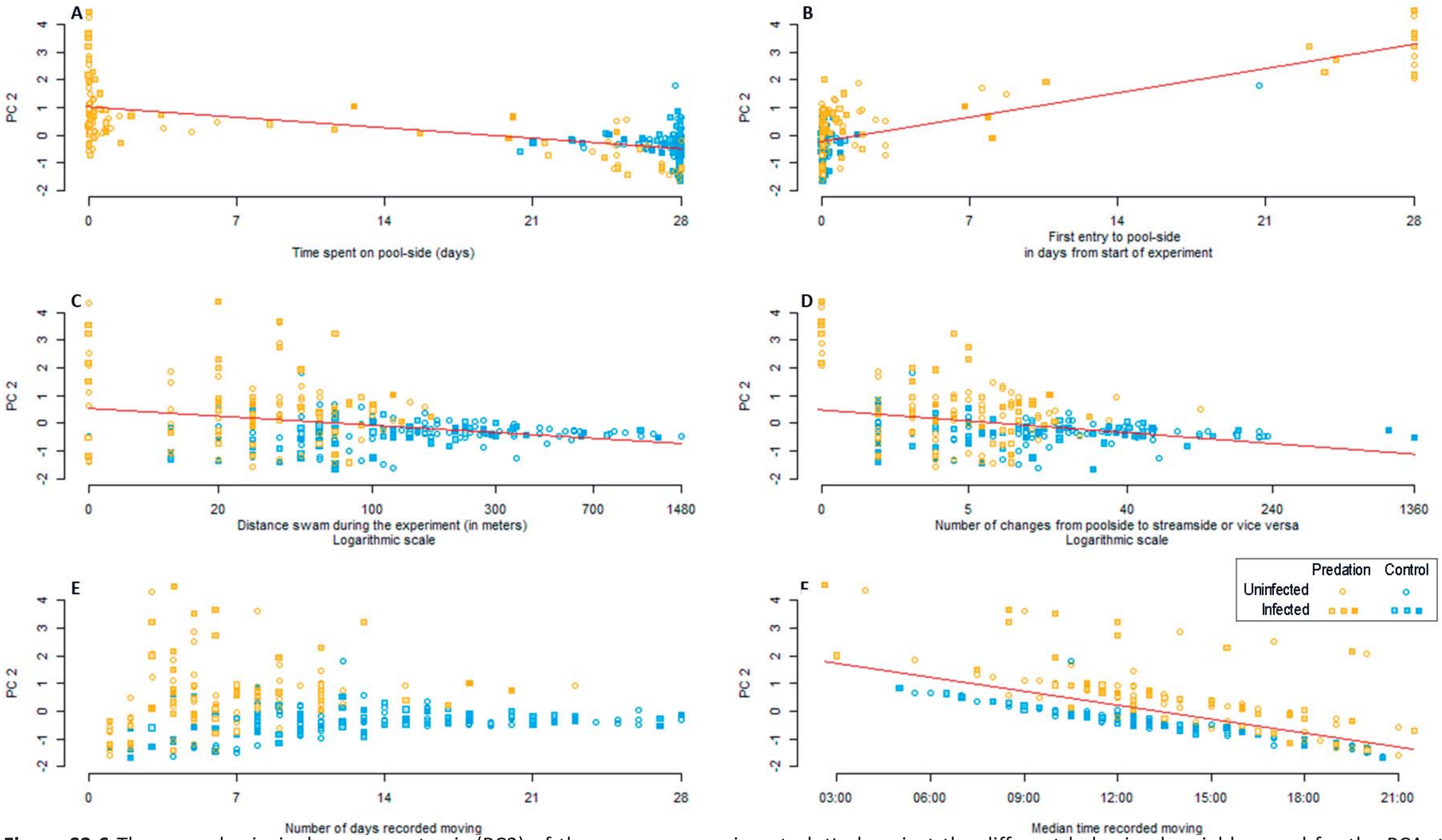

Figure S3.6 The second principal component axis (PC2) of the summer experiment plotted against the different behavioral variables used for the PCA: the proportion of time spent in the pool section of the tanks in days (a), the first entry to the pool-side section in days (b), the total distance swam during the experiment on a logarithmic scale in meters (c), the number of changes between the stream- and pool-sections on a logarithmic scale (d), the number of days on which an individual was recorded (e), and the median clock hour of the day the individuals were moving (f). Individuals in predation tanks are represented by yellow symbols and control tank individuals by blue symbols, where empty points represent uninfected individuals and squares represent infected individuals. Statistically significant correlations as red lines (p <0.001).
